# Controlled pathways and sequential information processing in serially coupled mechanical hysterons

**DOI:** 10.1073/pnas.2308414121

**Published:** 2024-05-20

**Authors:** Jingran Liu, Margot Teunisse, George Korovin, Ivo R. Vermaire, Lishuai Jin, Hadrien Bense, Martin van Hecke

**Affiliations:** ^a^Huygens-Kamerlingh Onnes Lab, Leiden Institute of Physics, Universiteit Leiden, NL-2300 RA Leiden, The Netherlands; ^b^Laboratory for Multiscale Mechanics and Medical Science, State Key Lab for Strength and Vibration of Mechanical Structures, School of Aerospace, Xi’an Jiaotong University, Xi’an 710049, China; ^c^AMOLF, 1098 XG Amsterdam, The Netherlands; ^d^Nonlinear Physical Chemistry Unit, Université Libre de Bruxelles, 1050 Bruxelles, Belgium

**Keywords:** mechanical metamaterials, hysterons, pathways, memory

## Abstract

The complex sequential response of frustrated materials results from the interactions between material bits called hysterons. Hence, a central challenge is to understand and control these interactions, so that materials with targeted pathways and functionalities can be realized. Here, we show that hysterons in serial configurations experience geometrically controllable antiferromagnetic-like interactions. We create hysteron-based metamaterials that leverage these interactions to realize targeted pathways, including those that break the return point memory property, characteristic of independent or weakly interacting hysterons. We uncover that the complex response to sequential driving of such strongly interacting hysteron-based materials can be described by finite state machines. We realize information processing operations such as string parsing in materia, and outline a general framework to uncover and characterize the FSMs for a given physical system. Our work provides a general strategy to understand and control hysteron interactions, and opens a broad avenue toward material-based information processing.

## Significance

Driven frustrated materials evolve via sequences of transitions between metastable states, associated with the flipping of one or more material bits called hysterons. Interactions between hysterons are key for obtaining and understanding complex pathways, yet their control has remained elusive. Here, we uncover a general geometric mechanism that yields controllable hysteron interactions. We characterize the resulting pathways and leverage our insights to fabricate metamaterials with targeted pathways that materialize counting, pattern generation, and pattern recognition. Our results open a route toward material-based sequential computing.

Frustrated media—crumpled sheets, disordered magnets, metamaterials, amorphous media—are generally multistable (1–7). The multiplicity of metastable states can be used to store information, and in many systems, each collective state can be encoded by the binary phase of localized “material bits” called hysterons—bistable elements such as rearranging particle clusters or snapping slender elements. Hence, information can be stored in the geometric configurations of these elements, which has been explored for, e.g., Braille displays (8), information storage (6, 9, 10) or shape changing objects (11). Here, we focus on the intermittent response to global driving of such complex media, which features pathways where the system hops between metastable states (1–4, 12–19). These pathways can be captured by a transition graph (t-graph), which fully characterizes the materials’ response to any driving protocol, and whose structure encodes, e.g., memory effects (1, 2, 5, 20–25). It has been recently shown that material pathways, where the system evolves between states, can be encoded and studied via the equivalent pathways of collections of interacting hysterons (20–24, 26). Surprisingly, t-graphs are reminiscent of the directed graphs that describe finite state machines (FSMs) (24), which are the essential models that describe sequential computation (27, 28). This similarity suggests that the ability to control and design targeted pathways would open a route toward advanced programmable matter (29, 30) and information processing in materia (31).

**Fig. 1.**
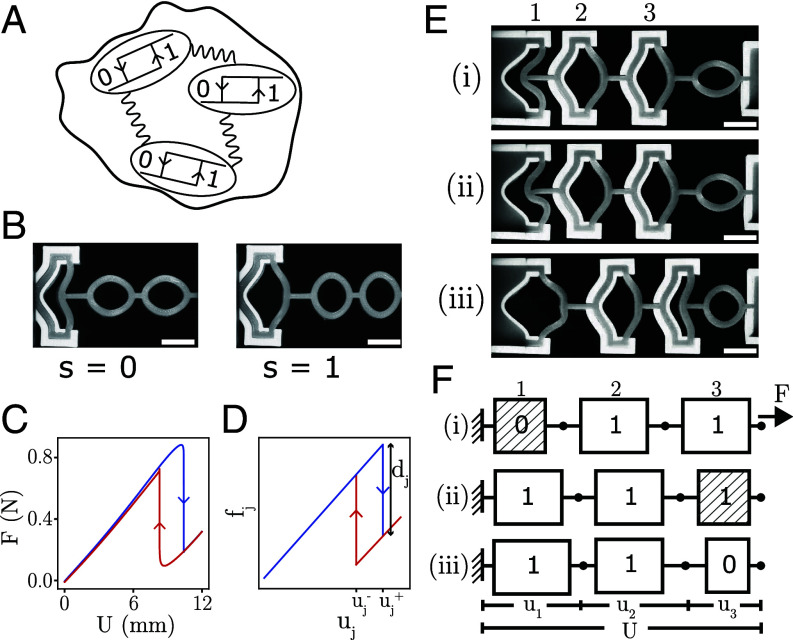
Hysterons and avalanches. (*A*) Bistable regions embedded in a material acting as interacting hysterons. (*B*) Experimental realization of a single hysteron, consisting of an encased, curved beam coupled in series with a spring (ellipses). *Left* and *Right* panels show the hysteron in phase s=0 and s=1 respectively. (Scale bar, 1cm.) (*C*) Experimental force–displacement curve. (*D*) Force–displacement curve of a bilinear hysteron, indicating the force drop $d_{j}$ and switching fields $u_{j}^{\pm }$. (*E*) Experiment showing three serially coupled mechanical hysterons which undergo an avalanche {011}→{110} when the total extensional deformation is increased (Scale bar, 1cm.) (*F*) Serially coupled mechanical hysterons satisfy force balance and a geometrical length constraint. Here, we illustrate the mechanism of the antiferromagnetic avalanche shown in panel (*E*): (i): Hysteron 1 becomes unstable (striped pattern); (ii): Hysteron 1 has switched $s_{1}:0\;\to \;1$ which causes it to extend, and which makes hysteron 3 unstable; (iii): Hysteron 3 has switched $s_{3}:1\;\to \;0$, after which the system equilibrates and all hysterons are stable.

Hysteron interactions, which physically are mediated by the passive background in which the hysterons are embedded (Fig. 1*A*), are crucial to realize a vast diversity of complex pathways and t-graphs (5, 24, 25). Without interactions, the pathways are very limited in diversity and scope, and in particular are constrained by Return Point Memory (RPM) (20, 23, 24), where the system returns to a previous state when a previous extremal driving is revisited; this leads to a specific “loop-within-loop” structure of the t-graphs (3, 20, 21). In the presence of (antiferromagnetic) interactions, a much wider variety of pathways and t-graphs have been observed, including pathways where the system evolves over multiple driving cycles and thus violates RPM (1, 5, 24, 25, 32, 33). However, we do not understand what sets the hysteron interactions and thus cannot control these. Experiments (1, 3, 34) and simulations (5, 24, 25, 35) have been mostly exploratory, without any strategy to realize targeted pathways or t-graphs. Controlling and fully exploiting the sequential response of complex materials thus requires to understand the physics of hysteron interactions and to leverage these insights to develop rational design of hysteron-based materials.

Here, we realize hysteron-based mechanical metamaterials with precisely controlled interactions and targeted pathways. We employ snapping beams as hysterons, mediate interactions via the elastic background through which the hysterons are coupled, and use the geometric beam parameters to control the hysteron parameters and interactions. We focus on serially coupled hysterons, and show that, as a direct consequence of the (mechanical) equilibrium conditions, these experience a specific form of global, antiferromagnetic interactions where the switching of one hysteron can trigger the switching of another element toward the opposite phase (24). For this class of interactions, we categorize all possible pathways and reveal that the corresponding t-graphs can be organized in a hierarchy, which simplifies their understanding and design. In particular, we find a wide variety of nontrivial pathways, not accessible in systems of independent hysterons, including those that break RPM and encode advanced functionalities.

Using our theoretical insights, we design and realize mechanical metamaterials that act as sequential pattern generators and which exhibit designable transient behavior in response to purely cyclical driving. We then show that the response of our metamaterials to more complex driving protocols, such as sequences of driving pulses with different amplitudes, encodes FSMs, and we leverage this insight to realize in-material sequential information processing. By controlling interactions and pathways, our work brings functional materials that execute precise sequences of configurational changes within reach, impacting, e.g., robotics and prosthetics. Furthermore, by encoding information in complex driving sequences, our “finite state materials” open a route toward computing in materia.

## 1. Models and Phenomenology

### 1.1. Mechanical Hysterons.

We consider n mechanical hysterons arranged along a chain. Each hysteron is based on the snapping instability (36, 37) of a flexible, precurved beam, encased in a rigid 3D-printed clamp. We connect the beam via a spring, consisting of one or more flexible elliptical elements, to an externally controlled actuator that sets the global deformation U (Fig. 1*B*; *Materials and Methods*). Measuring the extensional force F as function of U, we observe sharp, discontinuous, and hysteretic transitions between the “s=0” phase (beam curved leftward) and “s=1” phase (beam extended rightward) (Fig. 1 *B* and *C*). We note that at zero deformation, only the rest-state “0” is stable. The transitions are associated with sharp drops (0→1) or increases (1→0) of F, as well as nearly instantaneous changes in the beam configuration—this makes the experimental detection of transitions unambiguous. We define the upper and lower switching fields of hysteron j, $u_{j}^{+}$ and $u_{j}^{-}$, to correspond to the transitions $s_{j}:0\to 1$ and $s_{j}:1\to 0$ respectively. To simplify the theoretical analysis, we model the force–displacement relation of such mechanical hysterons by a bilinear relation between force $f_{j}$ and deformation $u_{j}$:

[1] $$\begin{array}{c} f_{j}=\left(u_{j}-d_{j}s_{j}\right)\;, \end{array}$$

where $d_{j}>0$ characterize the magnitude of the force discontinuities (Fig. 1*D*). We note that $u_{j}^{-}\;<\;u_{j}^{+}$ so that the beam is bistable for u in between these values. By varying the thickness and curvature of the beams we can control the values of the switching fields and force drops (*SI Appendix*).

### 1.2. Avalanches and Antiferromagnetic Interactions.

We now show that, due to the mechanical equilibrium conditions, serially coupled hysterons interact in an antiferromagnetic manner: The switching of one hysteron from phase 0 to 1 favors the switching of other elements from 1 to 0 (24). First, we experimentally demonstrate the existence of interactions by showing that three serially coupled hysterons can exhibit an avalanche, in which the phase change of one hysteron triggers the phase change in another (Fig. 1 *E* and *F*). We focus on a sample which initially is in state $S:=\left\{s_{1},s_{2},s_{3}\right\}=\left\{011\right\}$ (Fig. 1*E*—i). Increasing the global extension U, we observe that the leftmost (first) hysteron becomes unstable (Fig. 1*E*—ii) and transitions to its “1” phase; this immediately triggers hysteron three to switch from 1 to 0 (Fig. 1*E*—iii). We note that frames (ii) and (iii) are taken in quick succession, so that we associate the near-simultaneous switching of both hysterons with a single critical value of U where the collective state switches from {011} to {110}. We stress that hysteron three switches from phase 1 to 0, even though U is increased: this shows that the simultaneous switching of both hysterons cannot be due to a degeneracy of their respective switching fields, but is caused by antiferromagnetic interactions, where the switching of the first hysteron triggers a change in the conditions of the third hysteron that produces the avalanche. Hence, the observation of this avalanche unambiguously demonstrates the presence of antiferromagnetic hysteron interactions (24).

### 1.3. Mechanical Balance.

We now show that the hysteron interactions occur due to the mechanical balance conditions, and express these interactions in a recent model for interacting hysterons (5, 24, 25, 33). To intuitively understand how force balance and length constraints produce antiferromagnetic interactions in serially coupled hysterons, we consider the avalanche {011}→{110} again. When hysteron 1 transitions from 0→1, $u_{1}$ increases (Fig. 1*B*–*D*), and since the total displacement $U=u_{1}+u_{2}+u_{3}$ remains constant during the avalanche—the driving is quasistatic—the sum $u_{2}$+$u_{3}$ has to decrease. Consistent with this, the shape of the ellipse reveals that the force sharply drops (Fig. 1*E*). Hence, the switching of hysteron 1 lowers the force and extension of the other hysterons—this is what causes interactions—and in this particular case triggers the transition of hysteron 3 (Fig. 1*F*).

We make this notion precise by using our bilinear model for the hysteron mechanics (Eq. **1**). We assume quasistatic, overdamped dynamics, so that sweeping the global deformation U yields smooth episodes interspersed with discontinuous transitions (24). In each smooth episode, $\left\{s_{j}\right\}$ is constant, the hysterons are in mechanical equilibrium, and the local and global deformations and forces are related by a geometric length constraint and force balance (Fig. 1*F*):

[2] $$\begin{array}{cc} U & =\sum_{j}u_{j}\;. \end{array}$$

[3] $$\begin{array}{cc} F & =f_{i}=f_{j}\;\;\text{for all}\;i,j. \end{array}$$

Now consider the system being in state {011}, with the extension just reaching the critical value $U^{c}$ for hysteron 1 to become unstable (Fig. 1*F*—i). In our model, this forces the collective state to transition from S={011} to an intermediate state $S^{\prime }=\left\{111\right\}$ (Fig. 1*F*—ii). The deformations $u_{i}$ and force f in this intermediate state can be calculated using the geometric length constraint and force balance as $u_{1}^{\prime }=u_{1}+2d_{1}/3,u_{2}^{\prime }=u_{2}-d_{1}/3,u_{3}^{\prime }=u_{3}-d_{1}/3$, and $f^{\prime }=f-d_{1}/3$, where primed and unprimed variables refer to the state just after and before the transition (*SI Appendix*). Therefore, when hysteron 1 switches state the deformations of hysterons 2 and 3 suddenly drop—the amount of change depends on the value of the force drop and the number of hysterons—and their new values have to be compared to the lower switching fields of the two hysterons (since hysterons 2 and 3 are both in state 1). S^′^ is unstable if the displacement of hysteron 2 or 3 becomes lower than their lower switching fields. For our specific example, $u_{3}^{\prime }$ falls below $u_{3}^{-}$; hence, state $S^{\prime }$ is unstable and hysteron 3 transitions 1→0 (Fig. 1*F*—iii). After this additional transition we recalculate the deformations and force, compare each deformation to the corresponding switching fields, and if all hysterons are stable (as is the case here), we have obtained the full transition occurring at $U=U^{c}$ (*SI Appendix*).

### 1.4. Mapping to General Model.

To understand the interactions of serially coupled hysterons, we map their behavior to a recent, explicit, and general model of interacting hysterons (5, 24, 25, 33). In this general model, interactions are implemented by an explicit linear dependence of the global switching fields $V_{i}^{\pm }\left(S\right)$ on the collective state S (5, 24, 25, 33):

[4] $$\begin{array}{c} V_{i}^{\pm }\left(S\right)=v_{i}^{\pm }-\sum_{j}c_{\mathrm{ij}}s_{j}\;. \end{array}$$

Here, positive and negative $c_{\mathrm{ij}}$ encode, respectively, ferromagnetic and antiferromagnetic interactions, the self-couplings $c_{\mathrm{ii}}$ equal zero, and $v_{i}^{\pm }$ are the “bare” switching fields (24).

For serially coupled hysterons, using Eqs. **1**–**3**, we obtain the global state-dependent switching fields $U_{i}^{\pm }\left(S\right)$ in terms of the hysteron parameters as:

[5] $$\begin{array}{c} U_{i}^{\pm }\left(S\right)=n\left(u_{i}^{\pm }-d_{i}s_{i}\right)+\sum_{j}d_{j}s_{j}, \end{array}$$

which is equivalent to the general model using the mapping:

[6] $$\begin{array}{cc} V_{i}^{\pm } & =U_{i}^{\pm }/n, \end{array}$$

[7] $$\begin{array}{cc} v_{i}^{+} & =u_{i}^{+}, \end{array}$$

[8] $$\begin{array}{cc} v_{i}^{-} & =\left(u_{i}^{-}-d_{i}\right)+d_{i}/n, \end{array}$$

[9] $$\begin{array}{cc} c_{\mathrm{ij}} & =-d_{j}/n\;\;\text{for}\;i\neq j, \end{array}$$

[10] $$\begin{array}{cc} c_{\mathrm{ii}} & =0. \end{array}$$

For details, see *SI Appendix*.

This mapping clarifies the nature of the interactions of serially coupled hysterons and simplifies the study of transitions and avalanches. First, the coupling is mean-field-like and the interactions are global—the switching of hysteron j influences all other hysterons equally. Second, interactions are antiferromagnetic since $c_{\mathrm{ij}}\leq 0$. Third, the strength of the interactions is set by the force drops $d_{i}$ so that for small d, serially coupled hysterons map to nearly independent hysterons. Fourth, the interaction strength $c_{\mathrm{ij}}$ diminishes as 1/n—clearly the switching of one hysteron has only a minor effect in long chains. Hence, serially coupled hysterons experience a specific form of global, antiferromagnetic interactions due to the balance equations.

## 2. Pathways and Experimental Realizations

### 2.1. Simple sc-Graphs.

**Fig. 2.**
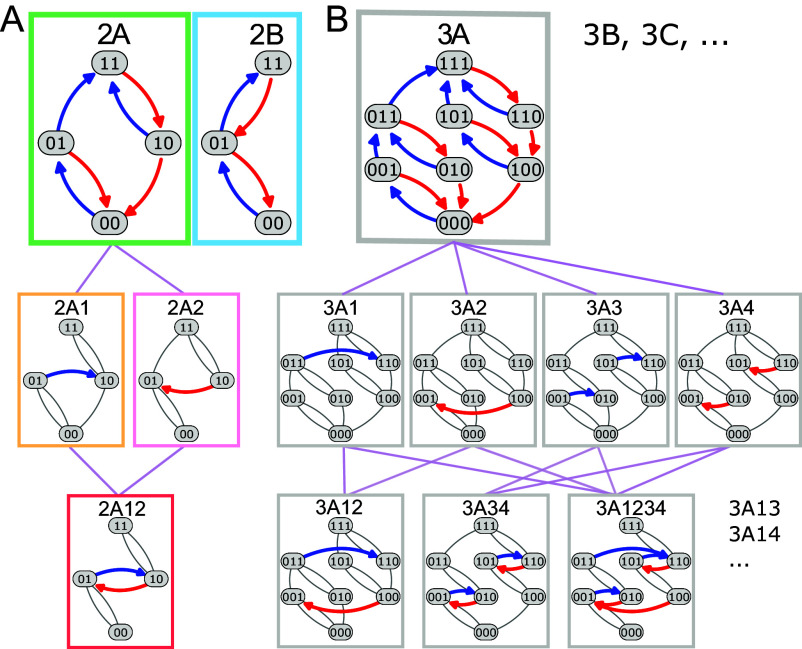
Diversity and hierarchical organization of simple sc-graphs. (*A*) Two hysterons produce two parent graphs, 2A and 2B. Graph 2A admits avalanches labeled 1 and 2, leading to the daughter graphs 2A1,2A2,2A12 [gray curves represent connections inherited from the P-graph while colored curves represent avalanches; for example, the avalanche {01}→{10} in 2A1 occurs by merging the transitions {01}→{11} and {11}→{10} from 2A (*SI Appendix*)]. The colors of the boxes correspond to those used in Fig. 3. (*B*) Three serially coupled hysterons feature six parent graphs 3A,3B,⋯, and 38 daughters. Shown here are graph 3A, all its first-generation daughters 3A1–3A4, and three additional daughters—for the complete hierarchy, see *SI Appendix*.

We have explored the space of attainable t-graphs for n serially coupled hysterons, using our mapping and a previously established numerical sampling algorithm (24) (*Materials and Methods*). To avoid complex situations featuring race conditions, we require that each elementary transition has only one unstable hysteron. We refer to the t-graphs for serially coupled hysterons with this additional requirement as simple sc-graphs, and find that they encompass both the Preisach graphs (P-graphs) that can also be found in the absence of interactions, as well as graphs that feature avalanches and which only can occur in the presence of interactions (Fig. 2). For example, there are five n=2 simple sc-graphs, two P-graphs, and three graphs containing avalanches; for n=3 we observe 44 distinct simple sc-graphs, six of which are P-graphs; for n=4, there are 550 simple sc-graphs of which 24 are P-graphs. For larger n, exhaustive sampling of the design space is challenging, but our data show more than 6,400 simple sc-graphs for n=5. Hence, sc-graphs are more numerous and varied than P-graphs.

For general interactions, the t-graphs can feature a wide variety of avalanches, and moreover, each state may have a different ordering of the switching fields, a phenomenon termed “scrambling” (1, 24) (*SI Appendix*). Even for three hysterons with general linear interactions, the full design space contains more than 15,000 t-graphs, and its organization is not well understood, hindering its exploitation and potential applications (24). In contrast, we find that simple sc-graphs have specific properties and can be organized in a simple hierarchical manner (Fig. 2). First, all avalanches in simple sc-graphs are of length two, and hence only have one intermediate state, and connect states with the same magnetization $m:=\Sigma_{i}s_{i}$—we refer to these as “horizontal avalanches.” Furthermore, simple sc-graphs do not allow scrambling, so that all sc-graphs without avalanches are P-graphs. Finally, simple sc-graphs do not feature orbits where two up or two down avalanches occur subsequently. For a rigorous derivation of these properties, see *SI Appendix*.

As a consequence, all nontrivial simple sc-graphs are obtained by modifying P-graphs by merging pairs of up and down transitions to form avalanches (Fig. 2). This leads to a hierarchical organization of the simple sc-graphs, where the parent graphs are the n! P-graphs which we label as nA,nB,⋯, and daughter graphs can be labeled by the presence of horizontal avalanches. For example, the five n=2 simple sc-graphs contain the parent graphs 2A and 2B and daughter graphs which contain one (2A1, 2A2) or two (2A12) avalanches (Fig. 2*A*). For n=3 there are n!=6 parent graphs (3A,3B,⋯), with their 38 daughter graphs featuring between one and six avalanches—a few examples of these are shown in Fig. 2*B* (see *SI Appendix* for full hierarchy and details). Hence, serially coupled hysterons feature specific interactions that allow to fully understand and classify their corresponding t-graphs, and this facilitates the systematic exploration of all accessible pathways.

Crucially, serially coupled hysterons can violate RPM, which is essential for allowing a complex response, such as transients. RPM is the widespread ability of complex systems to “remember their extremal driving,” i.e., to return to a previously visited state when the driving revisits an extremum value (12, 32, 38, 39). A subtle breaking of RPM is possible for any simple sc-graph, depending on the precise values of its switching fields (1). In addition, several simple sc-graphs have a topology that strongly breaks RPM, regardless of the values of the switching fields. Strong RPM breaking can be tested via so-called loop-RPM (l-RPM)—informally, l-RPM requires that any sequence of state transitions forming a loop is escaped by going through either the top or bottom state of this loop, and cannot be escaped via an intermediate state (For a formal definition see *SI Appendix*) (1, 20, 21). Seven of the n=3 simple sc-graphs break l-RPM, including the graphs 3A1,3A2 and 3A12 shown in Fig. 2*B*. For larger n, we find that the fraction of simple sc-graphs that break l-RPM are ≈0.34 for n=4 and ≈0.42 for n=5. Hence, serial interactions open a broad avenue toward realizing interacting hysteron systems with complex pathways.

### 2.2. Realization and Design Space.

**Fig. 3.**
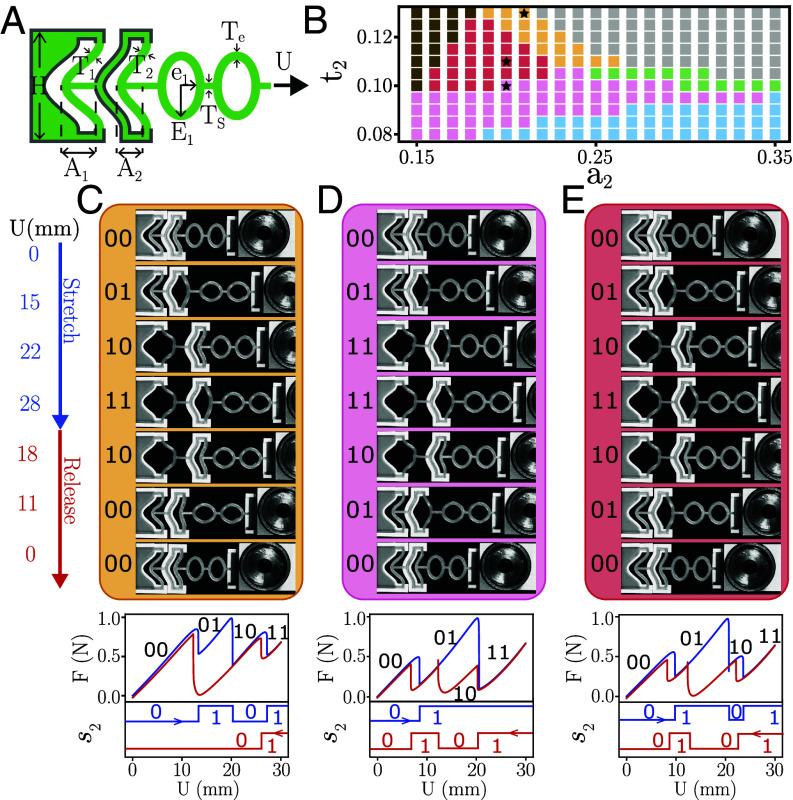
Two serially coupled mechanical hysterons. (*A*) Geometry with two encased curved beams with dimensionless thickness $t_{i}=T_{i}/H$ and dimensionless amplitude of the profile of the precurved beam $a_{i}:=A_{i}/H$, coupled to a spring [elliptical units (*Materials and Methods*)]. (*B*) Design space. We fix the parameters of hysteron 1 at $a_{1}=0.4$, $t_{1}=0.1$, and of the spring (*Materials and Methods*), and numerically investigate the t-graph type as function of the design parameters of hysteron 2. We find all five types of sc-graphs (color corresponding to Fig. 2*A*). While we focus on the parameter range where $\left\{00\right\}\overset{\mathrm{up}}{\to }\left\{01\right\}$, for large $a_{2}$ and $t_{2}$ we also find cases where $\left\{00\right\}\overset{\mathrm{up}}{\to }\left\{10\right\}$ - such t-graphs are related to our target graphs by relabeling symmetry of the hysterons (gray region). For small $a_{2}$ and large $t_{2}$, the transitions lose their sharp and hysteretic nature (brown region). Stars indicate the design parameters used in *C*–*E*. (*C*–*E*) *Top*: Main loops of experimental realizations of two serially coupled hysterons that materialize t-graphs 2A1, 2A2, and 2A12 (*Materials and Methods*). *Middle*: Corresponding force–displacement curves with states labeled. *Bottom*: The phase of hysteron 2, $s_{2}$, during up (blue) and down (red) ramps illustrates pattern generation.

We now aim to realize hysteron-based metamaterials with controlled interactions and pathways, starting with two-hysteron systems (Fig. 3). Since the mapping between design parameters ($a_{i},t_{i}$), respectively the normalized amplitude of the profile and thickness of the precurved beam (Fig. 3 and *Materials and Methods*), and hysteron parameters is complex, we first perform a systematic scan of the design space by finite element simulations with the commercially available FEM software ABAQUS. We fix the design parameters of hysteron 1, scan the design parameters $t_{2}$ and $a_{2}$ of hysteron 2, and obtain the corresponding t-graphs via extension-compression sweeps of U. Within this two-dimensional design space, all five n=2 simple sc-graphs can be realized (Fig. 3*B*). We target the nontrivial simple sc-graphs 2A1, 2A2, and 2A12, select design parameters based on our simulations, and fabricate three experimental two-hysteron systems that we find to realize the pathways corresponding to these nontrivial simple sc-graphs (*Materials and Methods*).

Cyclic driving of these samples illustrates their pattern-generating properties (Fig. 3*C*–*E*). Specifically, while noninteracting hysterons only flip phase once during a monotonous driving ramp, in our samples hysteron 2 changes phase twice (e.g., 0→1→0→1) during either the up ramp (2A1), down ramp (2A2), or both ramps (2A12), as a result from the avalanches that connect states {01} and {10}. As a consequence, these samples visit all four states while driven monotonically, and act as two-bit binary counters. Hence, these metamaterials act as pattern generators that translate continuous ramps into sequential patterns, which may feature distinct patterns on the up and down sweep, and which could be used to generate, e.g., the stride patterns of the legs of a soft robot (16, 40, 41).

### 2.3. Complex Pathways.

To obtain a metamaterial with orbits that break RPM, we require at least three hysterons, and out of all n=3 simple sc-graphs that break l-RPM, we target simple sc-graph 3A12—which breaks l-RPM in two different manners—and investigate the nature of the ensuing complex pathways. The probability to obtain this simple sc-graph in random sampling of the coupled hysteron model is small, of the order $1.2\times 10^{-3}$. To rationally design an experimental sample, we note that “freezing” one of the hysteron phases produces n=2 subgraphs, e.g., fixing the first hysteron produces subgraphs for hysteron two and three of type 2A. Our numerical design rules (Fig. 3*B*) suggest ratios for pairs of the design parameters $a_{1}$-$t_{3}$ from which we obtain a range of suitable design parameters. We realize four samples A–D within this design space, experimentally determine their full transition graphs, and find that all four materialize the target simple sc-graph 3A12 (*SI Appendix*). In the remainder, we focus on the behavior of sample A (for design parameters, see *Materials and Methods* and *SI Appendix*).

**Fig. 4.**
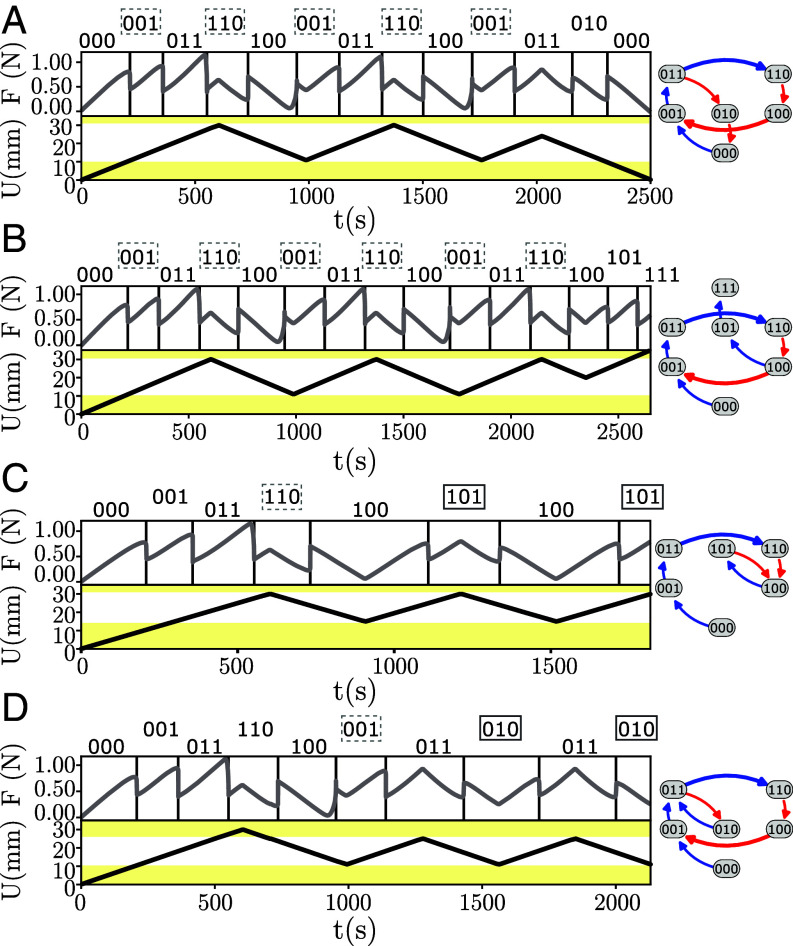
Nontrivial pathways (Movies S1–S4). Subjecting sample A which is described by simple sc-graph 3A12 to cyclic driving, we observe two pathways that break l-RPM, and two pathways that constitute a τ=2 transient. *Left*: Sweeping U(t) as indicated produces F(t) whose discontinuities indicate transitions. Each state (indicated above the graph) is obtained by inspecting the real-space configuration of the system. *Right*: relevant part of the simple sc-graph. (*A* and *B*) The loop between {001} and {110} (dashed boxes) is escaped by reversing the sweep direction during the up ramp (*A*) or down ramp (*B*), demonstrating (l-)RPM violations. (*C*) Starting from U=0 mm and S={000}, and repeatedly cycling U to 30mm, state {110} (dashed) is reached at the first maximum, and {101} (box) at subsequent maxima. (*D*) Starting from U=25mm and S={110}, and then repeatedly cycling U down to 11mm, state {001} (dashed) is reached at the first minimum, and {010} (box) at subsequent minima.

We now demonstrate that our sample breaks l-RPM in two different manners. We first cycle U between appropriate extremal values $U_{m}$ and $U_{M}$ to reach the loop $\left\{001\right\}\;\overset{\mathrm{up}}{\to }\;\left\{011\right\}\;\overset{\mathrm{up}}{\to }\;\left\{110\right\}\;\overset{\mathrm{down}}{\to }\;\left\{100\right\}\;\overset{\mathrm{down}}{\to }\;\left\{001\right\}$. We then reverse the driving direction at an intermediate value of U (Fig. 4 *A* and *B*). Systems that satisfy l-RPM would revisit one of the extremal states {001} or {110} before escaping the loop, but our experiments show that our sample escapes this loop, either in the up or the down direction, without revisiting the extremal states (Fig. 4 *A* and *B*). These orbits directly demonstrate violations of l-RPM, and thus RPM (1, 21).

Systems that violate l-RPM can exhibit nontrivial transients (1, 25). We find that the response of our sample to cyclical driving, for appropriate values of $U_{m}$ and $U_{M}$, indeed produces such a transient response: the system reaches its minimal or maximal state not in the first but only in the second (and subsequent) driving cycles which we call an accumulator behavior. (Fig. 4 *C* and *D*). Our three-hysteron experimental system thus implements an accumulator, or counter (1).

## 3. Sequential Information Processing and Finite State Materials

We now uncover a powerful connection between the sequential response of our metamaterials and FSMs (1, 24, 27, 28). While t-graphs conveniently describe the orbit, i.e., sequence of states, for a given driving protocol and initial state, they are not well-suited for addressing “inverse” questions such as: Which driving protocols lead to a specific orbit? What are the possible transients under cyclic driving, and what are their statistics? For which values of $U_{m}$ and $U_{M}$ can the longest transients be observed? What is the response to sequences of driving pulses? As we show below, mapping the combination of a t-graph and a protocol—encoded in an alphabet—to an FSM, allows to answer such questions. In particular, such FSMs allow to map a continuous search in the multidimensional space of driving protocols to a discrete search in the space of driving characters and strings.

First, we introduce our approach by three concrete examples that exemplify how the sequential response of our metamaterials can be described by an FSM. Second, we introduce a general framework for systematically constructing driving alphabets, based on the key observation that, for finite systems, driving protocols can be discretized. Third, we use FSMs to effectively characterize transient responses under cyclic driving. Finally, we systematically explore the response to sequences of pulses, and use the corresponding FSMs to characterize the computational complexity and memory capability of systems described by t-graphs. Together, our approach establishes a systematic framework to capture and explore the sequential response of any system described by a t-graph.

### 3.1. Sequential Computations with Sample A.

We start by illustrating the connection between sequential response and computations for sample A. We define a complex driving protocol, consisting of sequences of pulses, that serves as the input to the metamaterial. We then show that its response and final state are complex functions of the pulse sequence, and interpret these functions in the language of FSMs (1, 24, 27, 28). FSMs are the paradigmatic model of sequential information processing with memory (27, 28). In essence, an FSM is defined by finite set of states, a finite set of input characters called the alphabet, and a transition table which lists how the state changes in response to input. Furthermore, it is customary to define an initial state, one or more final or “accepting” state, and characterize the FSM by the collection of input sequences that take the system from the initial to an accepting state (27, 28). Despite their simple definition, their computational power is qualitatively and vastly larger than that of combinational logic (42–44), and in fact describes computing in any finite machine (27, 28). Realizing FSMs in materia is hence a crucial step toward material-based computation.

To illustrate that multiple FSMs are embedded in physical systems described by t-graphs, we introduce driving pulses where we quasistatically sweep the driving U as $U_{0}\to U_{a,b,\cdots }\to U_{0}$ for given choices of $U_{0},U_{a},U_{b},\cdots$—each such pulse is then associated with a character a,b,⋯. For this protocol, the states of the FSM are given by the states $S_{s}=\left\{S_{0},S_{1},\cdots \right\}$ that are stable at $U=U_{0}$. Since the t-graph completely determines the response of the system to any driving, it yields a transition table that maps any state in $S_{s}$ and character to a product state in $S_{s}$ — thus defining an FSM.

**Fig. 5.**
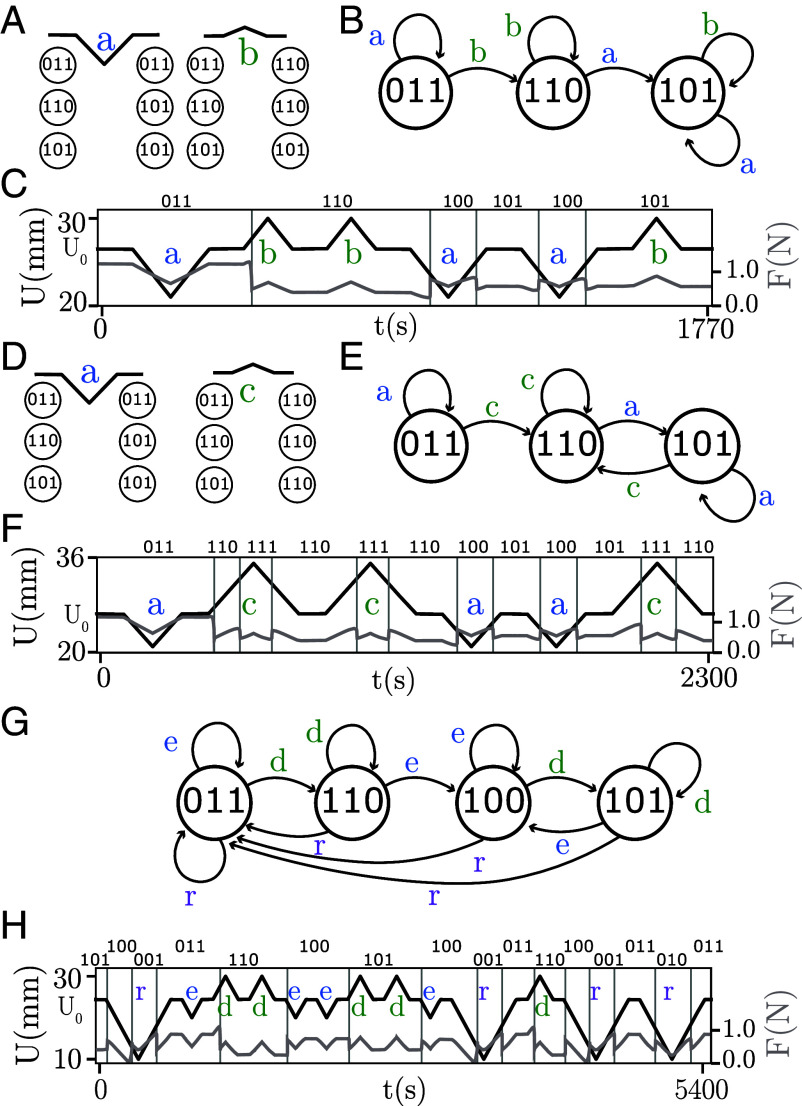
Sequential input and FSMs (Movies S5–S7). All data is for Sample A; for precise definitions of all peaks, see text. (*A*) The driving pulses a and b map states (*Left*) to product states (*Right*). (*B*) State transition diagram. (*C*) Global force F as function of U shows all possible state transitions; binary states are determined from real space images. (*D* and *E*) Transition table and diagram for driving pulses a and c. (*F*) Force F and states for a and c pulses. (*G*) State diagram for the three pulsed d, e, and r, and (*H*) corresponding forces and states.

We now illustrate the experimental realization of FSMs in our samples for three choices of our alphabet, i.e., choices of driving pulses. For the first FSM, we take $U_{0}=26.5$ mm, $U_{a}=21$ mm and $U_{b}=30$ mm. Therefore, $S_{s}$ consists of the three states {011},{101}, and {110}, and we define {011} and {101} as starting and accepting states. The state transitions in FSM1 follow from the response to the pulses a and b predicted by the t-graph: $S_{0}\;\overset{a}{\to }\;S_{0}$, $S_{1}\;\overset{a}{\to }\;S_{2}$, $S_{2}\;\overset{a}{\to }\;S_{2}$, $S_{0}\;\overset{b}{\to }\;S_{1}$, $S_{1}\;\overset{b}{\to }\;S_{1}$ and $S_{2}\;\overset{b}{\to }\;S_{2}$ (Fig. 5*A*). The corresponding FSM 1 reaches the accepting state for driving sequences which first contain an arbitrary number of a, then at least one b, and then at least one a, such as ba, abba, and aaabbabbab (Fig. 5*B*). We probe the response of sample A to sequential input, and find that it fatefully executes all FSM transitions, demonstrating the experimental realization of FSM 1 (Fig. 5*C*, *SI Appendix*, and Movie S5). We stress that while all accepted input sequences contain at least one a and one b, FSM1 is distinct from an Abelian memory, i.e., sequence-independent memory of inputs a and b: FSM 1 does accept ba but not ab.

Second, to demonstrate that the same sample encodes a range of FSMs, we define $U_{c}=35$ mm and consider the alphabet {a,c} which yields FSM 2 (Fig. 5 *D* and *E*). Crucially, the accepting state {101} is not absorbing, as $\left\{101\right\}\overset{c}{\to }\left\{110\right\}$. Therefore, FSM 2 accepts input strings that start with an arbitrary number of a, then contain at least one c, and then a sequence of a and c ending on a; FSM 2 accepts “acaa” but not “acac” (Fig. 5*F*, Movies S6, and *SI Appendix*).

Third, we consider a more complex FSM 3 where four states ({011},{110},{100} and {101}) are stable for $U_{0}=24.4\;$mm. We define three characters r, d and e with $U_{r}=10$ mm, $U_{d}=30$ mm, and $U_{e}=20$ mm; r acts as a reset that brings the system to the starting state {011} (Fig. 5*G*). To reach the accepting state {101}, the input string, after an arbitrary number of r and e, should contain no r, at least one d, then at least one e, and then an arbitrary combination of d and e ending on d; FSM3 accepts “ded,” “derded,” “deded,” but not “dede,” “dedr,” or “dredee”—in other words, reaching state {101} encodes a complex memory of the driving protocol, requiring at least the three pulse sequence (ded) (Fig. 5 *G* and *H*, Movies S7, and *SI Appendix*).

Together, these three examples illustrate how the sequential response of a physical system of interacting hysterons can be described by FSMs and that even relatively simple systems and driving protocols can reveal significant memory effects.

### 3.2. Driving Protocols, Characters, and Alphabets.

Appropriate driving protocols allow to access specific properties, e.g., cyclic driving is useful to characterize transients, whereas pulse driving can be useful to probe memory and computations. The choice of driving protocol translates to a choice of the alphabet, and is crucial to translate a physical system or t-graph to a specific FSM: different alphabets yield different FSMs. This difference reflects that a full understanding of the response under one protocol (e.g., cyclic driving) does not trivially translate to the response under another (e.g., sequences of driving pulses). Here, we introduce a general framework for systematically constructing driving characters and alphabets.

**Fig. 6.**
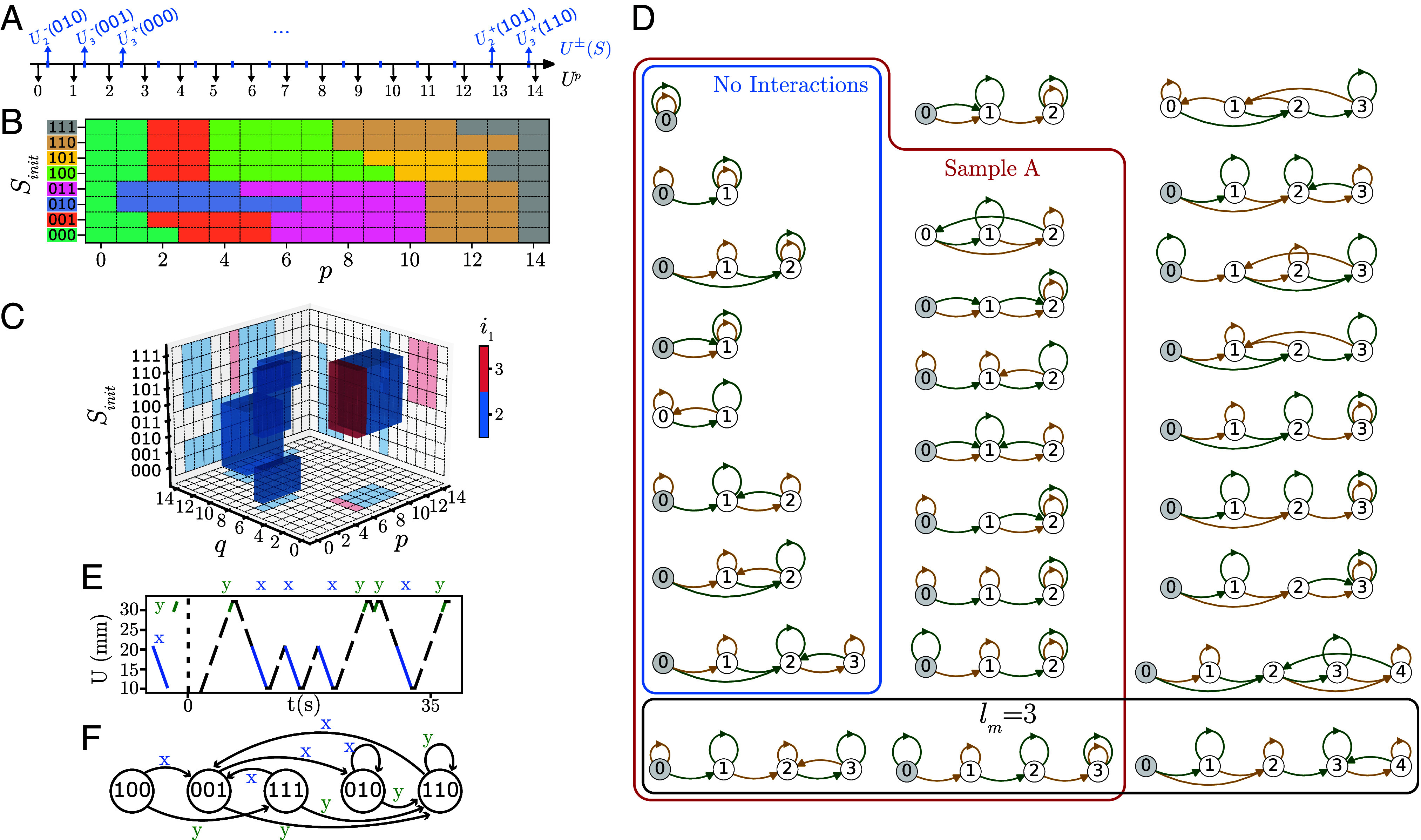
Discretization, computational power, and memory. (*A*) Illustration of the construction of the representative values $U^{p}$ for sample A, which has 14 switching fields leading to 15 representative values $U^{0}-U^{14}$. (*B*) Transition table $x_{p}$ for sample A—note that this table is distinct from the transition tables of the t-graph. (*C*) Measure of the transient duration $i_{1}$ for sample A, as function of initial state and driving amplitude labels p and q. (*D*) The 27 irreducible FSMs found for n=3 serially coupled hysterons. These include the eight FSMs for noninteracting hysterons (blue box); the 17 FSMs for sample A (red box); and three FSMs with maximal shortest path $l_{m}=3$ (black box). The initial states are indicated in gray—when there is no gray node, all states can serve as initial states. (*E* and *F*) Driving protocol based on a two character alphabet of ramps, and corresponding FSM for sample A.

First, we note that driving protocols can be broken up in discrete, elementary operations. In the quasistatic limit, any driving protocol consists of a sequence of up and down sweeps of U and is specified by a sequence of extrema (minimal and maximal driving amplitudes) $\left[U_{\mathrm{ex}}\left(0\right),U_{\mathrm{ex}}\left(1\right),\cdots \right]$. Crucially, small modifications of $U_{\mathrm{ex}}\left(i\right)$ do not modify the response when they do not cross any of the switching fields. As the number of switching fields is finite (for finite systems), the driving amplitudes and corresponding elementary up and down sweeps can be discretized. We illustrate this discretization for the example of sample A. We order the 2N−2 values of the switching fields $U^{\pm }\left(S\right)$ and define 2N−1 representative values $U^{p}$ for each interval between subsequent switching fields (Fig. 6*A*). This allows to map any driving protocol to a countable set of discretized protocols $\left[U^{p_{0}},U^{p_{1}},\cdots \right]$, where $p_{i}$ are integers. To capture the response, we define a discrete set of elementary operations $x_{p}$ as the response when U is quasistatically swept to $U^{p}$. We relax the tacit condition, used for FSM 1–FSM 3 (Fig. 5), that driving protocols start within the range of stability of state S. Instead, when $U^{p}$ is outside this range, we add an initial quasistatic ramp of U from the stable range to $U^{p}$. This allows to determine the transition table $S^{\prime }=x_{p}\left(S\right)$ for any combination of p and S (Fig. 6*B*).

Second, any set of distinct characters, i.e., any alphabet, can be composed from these elementary operations. Here, we give two examples that we will use below to study, respectively, transients and computations. First, we define the trivial alphabet $\Sigma_{0}:=\left\{x_{p}\right\}$. While all driving protocols can be encoded in $\Sigma_{0}$, the corresponding FSMs are limited, since $x_{p}\;○\;x_{p}\left(S\right)=x_{p}\left(S\right)$ for any character $x_{p}$ (here ○ denotes functional composition). Strikingly, while protocols based on cyclic driving or sequences of driving pulses restrict the possible sequence $\left[U^{p_{0}},U^{p_{1}},\cdots \right]$, such alphabets can lead to more complex FSMs. A prime example is the alphabets composed of driving pulses $\Sigma_{1}^{p}:=\left\{a_{p}^{q}\right\}$, where U is swept as $U^{p}\;\to U^{q}\;\to U^{p}$. These pulses $a_{p}^{q}$ are compounds of the elementary operations: $a_{p}^{q}\left(S\right):=x_{p}\;○\;x_{q}\;○\;x_{p}\left(S\right)$—note that the alphabets of FSMs 1–3 are subsets of $\Sigma_{1}^{p}$. (Subsets of) these alphabets lead to more complex FSMs, and in particular, we find FSMs where $a_{p}^{q}\;○\;a_{p}^{q}\left(S\right)\neq a_{p}^{q}\left(S\right)$ (see below). We stress that uppercase quantities like U always refer to actual values of the driving field, while lowercase quantities like $a_{p}^{q}$ refer to symbolic operators.

### 3.3. Transient Behavior.

As a first example of the utility of the FSM framework, we characterize the transient behavior under cyclic driving, where U is cycled between $U_{m}$ and $U_{M}$. We study orbits starting from $S_{\mathrm{init}}$ under cyclic driving $S_{\mathrm{init}}\overset{x_{p}}{\to }S_{0}\overset{x_{q}}{\to }S_{1}\overset{x_{p}}{\to }S_{2},\cdots$, where $x_{p},x_{q}\in \Sigma_{0}$. As the number of states is finite and the system is deterministic, the orbit becomes periodic, and we denote the first and second index of the first repeating state as $i_{1}$ and $i_{2}$ respectively: for the example shown in Fig. 4*C*, where $S_{\mathrm{init}}=\left\{000\right\}$, the orbit $\left[S_{0},S_{1},S_{2},\cdots \right]=\left[001,110,100,101,100,101\right]$ yields $\left(i_{1},i_{2}\right)=\left(2,4\right)$.

To determine the range and statistics of transients, one could in principle sample $U_{m},U_{M}$ and the initial state (3, 5, 25). However, this method, based on random sampling lacks efficiency. The FSM framework offers a more effective tool as it uses a finite number of calculations, and we straightforwardly determine $i_{1}\left(p,q,S_{\mathrm{init}}\right)$ and $i_{2}\left(p,q,S_{\mathrm{init}}\right)$ (Fig. 6*C*). The FSM framework captures all possible orbits, transients, and periods, including rare behaviors that occur in narrow intervals between specific switching fields and facilitates the straightforward calculation of statistical measures such as the average transient time, by translating the pertinent ranges of p and q to volumes in $U_{m},U_{M}$ space using the intervals corresponding to $U^{p}$ and $U^{q}$ (Fig. 6*A*). Hence, the finite alphabets allow to turn explorations of the multidimensional continuous space of driving protocols into discrete, combinatorial explorations. For details, see *SI Appendix*.

### 3.4. Computational Power and Memory Capacity.

To gain insight into the computational capabilities of serially coupled hysterons, we systematically extract their FSMs under driving alphabets consisting of two pulses, and introduce measures of their memory capacity and computational capability. We compare the FSMs of sample A to those of noninteracting hysterons, serially coupled hysterons, and generally interacting hysterons.

To determine all two pulse FSMs for a given t-graph, we scan over the three-parameter family of two-character alphabets $\left\{a_{p}^{q_{1}},a_{p}^{q_{2}}\right\}$, and for each initial state, stable at $U^{p}$, determine candidate FSMs. Many of these are equivalent under permutations of the characters $a_{p}^{q_{1}}↔a_{p}^{q_{2}}$ or the (noninitial) states. Focusing on the irreducible FSMs which remain distinct under such permutations, and labeling the states of the FSM as 0,1,⋯, thus ignoring the hysteron phases, we find that sample A allows for 17 distinct FSMs (Fig. 6*D*). For details, see *SI Appendix*.

To characterize these FSMs, we note here a similarity between these FSMs and notions of memory in complex materials (22). In material memory, one prepares a physical system in a certain (ensemble of) state(s); then drives the system; and then asks what information the final states encode about the driving (22). For finite, deterministic, quasistatic systems described by t-graphs, this question is tantamount to asking which strings of characters bring a system from an initial state to a final state.

This notion suggests two simple characterizations of the FSMs. First, the size (number of nodes) of the FSM can serve as a simple measure of storage capacity, and for sample A, varies between one and four (Fig. 6*D*). Second, if the system is prepared in state $S_{i}$, and after driving reaches state $S_{j}$, we can infer that $S_{j}$ encodes a memory of at least $l(S_{i},S_{j})$ pulses, where $l(S_{i},S_{j})$ denotes the shortest path lengths from $S_{i}$ to $S_{j}$. We use the maximal shortest path, $l_{m}:=max_{\left\{S_{i},S_{j}\right\}}(l\left(S_{i},S_{j}\right))$ to capture this memory capacity. For sample A, appropriate choices of the input pulses can produce a capacity $l_{m}=3$—note that one of the corresponding FSMs is equivalent to FSM3 restricted to inputs d and e (Figs. 5*G* and 6*D*).

We now briefly discuss the multiplicity, size, and capacity $l_{m}$ of FSMs for ensembles of hysterons. For n=3 noninteracting hysterons i.e., the n=3 Preisach model, we only find eight FSMs, with maximal size four and $l_{m}\leq 2$; strikingly, for larger ensembles of noninteracting hysterons, we find the same eight FSMs. We can understand the occurrence of these eight FSMs by considering the action of sequences of positive and negative pulses on collections of independent hysterons (*SI Appendix*).

Ensembles of n=3 SC hysterons produce 27 distinct FSMs with maximal size five and $l_{m}$ up to three (Fig. 6*D*). These FSMs and their characteristics precisely specify the computational applications of three serially coupled hysterons. Explorations of ensembles of n=3 hysterons with general interactions (Eq. **4**) produce many more FSMs with sizes up to seven and $l_{m}$ up to six; for n>3, serially coupled hysterons also produce more FSMs of larger sizes and memory capacity.

These explorations do not exhaust the possible FSMs that can be realized, even for a given system: different alphabets of more or different components can yield an even wider variety. $\Sigma_{1}^{p}$ is restricted to pulse sequences which always return to driving amplitude $U^{p}$. We have therefore explored the alphabet $\Sigma_{2}:=\left\{r_{p}^{q}\right\}$, where the characters $r_{p}^{q}$ denote quasistatic driving ramps—if need be preceded by a quasistatic ramp of U from the stable range of S to $U^{p}$—and where $r_{p}^{q}\left(S\right):=x_{q}\;○\;x_{p}\left(S\right)$. We illustrate the use of ramp characters by combining sample A with an alphabet of two ramps $x:=r_{2}^{1}$ and $y:=r_{3}^{4}$, where $\left\{U^{1},U^{2},U^{3},U^{4}\right\}=\left\{10.2,20.9,29.6,32.2\right\}$mm. Any input string combining the characters x and y, such as “yxxxyyxy” can be translated to a specific driving protocol (Fig. 6*E*). The combination of the alphabet {x,y} and the t-graph of sample A produces FSM 4 (Fig. 6*F*; see *SI Appendix* for experimental data). We stress that FSM 4 is distinct from any of the FSMs shown in Fig. 6*D*, indicating the utility of specific alphabets to realize complex FSMs.

We finally show that for any FSM a corresponding t-graph and alphabet that realizes this FSM can be constructed. We consider a FSM with N states and L characters, and a transition table $z_{i}\left(S_{j}\right)=S_{k}$. To find a physical implementation, we encode the abstract characters $z_{i}$ as driving pulses, starting and ending at $U=U^{0}$, and with increasing peak amplitudes $U^{i}$, i.e., using the alphabet $\Sigma_{1}^{0}:=\left\{a_{0}^{i}\right\}$. Then we construct a t-graph by defining N×L states: N “ground states” $S_{j}^{0}$, and N×(L−1) “excited states” $S_{j}^{i}$. The states $S_{j}^{0}$ are the only states stable at U=0 and can be directly mapped to the nodes of the FSM. To implement the transition table $z_{i}\left(S_{j}\right)=S_{k}$, we construct the t-graph such that the upsweep of a driving pulse $a_{0}^{i}$ drives the ground state $S_{j}^{0}$ to the excited states $S_{j}^{i}$; and that the down sweep drives the excited state $S_{j}^{i}$ to the appropriate ground state $z_{i}\left(S_{j}\right)$. This general construction shows that t-graphs encode the same computational power as FSMs. For details, see *SI Appendix*.

## 4. Conclusion and Outlook

Our work reveals that (mechanical) equilibrium produces global, antiferromagnetic interactions in serially coupled hysterons. We have determined all simple sc-graphs for small groups of hysterons and derived general constraints on their type of avalanches and topology. We obtained design guidelines to materialize specific t-graphs, and use these to realize metamaterials that generate sequential patterns, act as 2-bit binary counters, and perform input string parsing. We showed more generally that the sequential response of (hysteronic) materials can be described by FSMs. This opens a route to effective calculations of statistical properties of the sequential response of complex materials, as well as providing insight into their memory and computational capabilities—such FSMs are a rich avenue for future research.

Our work has the potential to impact a broad range of hysteron-based systems. First, we stress that interactions governed by balance equations are not restricted to mechanics—e.g., similar interactions may arise in hysterons describing antibiotic resistance evolution (45), and electronic hysterons switching in response to an external voltage and coupled in series so that all currents must match would feature the same interactions (46). While mechanical implementations so far are large, slow, and energetically costly, the universality of hysterons and the particular interaction mechanism uncovered here may allow the development of hysteron-based computation that is ultrafast, miniaturized, and energy effective (46). Second, we note that the interactions studied here arise because we control the global deformations; if we control the global force of serially coupled hysterons, there are no interactions, which suggests that mixed boundary conditions allow to tune the interaction strength. Third, other embedding geometries can be used to tune the interactions. For example, we expect that for a linear configuration of hysterons embedded in a two-dimensional background, the interactions become localized, with the interaction distance tunable by geometry; parallel configurations may give rise to ferromagnetic interactions. Our work brings an understanding of the nature of interactions, as well as their rational design, within reach (1, 3, 5).

Important future questions include to understand the growth of the computational complexity with, e.g., the number of hysterons, type of interactions, and driving protocols, to link these FSMs to physically relevant properties such as RPM, l-RPM, transients, and subharmonic behavior, where under cyclic driving the system revisits earlier states only after more than one driving cycle, and more widely to understand the organization, limits, and design of these FSMs. In particular, increasing the number of hysterons pairs an explosion in possible FSMs to an increased sensitivity to noise and (plastic) aging effects that practical designs need to balance. We suggest that exploring designs with a finite range of interactions allows to create hierarchical structures where multiple small functional units robustly operate in parallel.

We end with two challenges that are key for realizing complex hysterons-based materials for in materia computing. First, although we have shown that any FSM can be embedded in a t-graph, the problem of designing a set of interacting hysterons for a given FSM is at present open. We have an explicit implementation of this construction for an arbitrary number of states and L=1; although we expect this construction to generalize to arbitrary L, this is left for future work (*SI Appendix*). Second, there is yet no precise design procedure to realize (meta)materials with hysterons with arbitrary interactions, although we expect that generalizations from a linear geometry to more complex 2D geometries may be sufficient. Both challenges seem surmountable, thus suggesting a concrete strategy to embed sequential computations in materia.

## 5. Materials and Methods

### 5.1. Geometry.

Our hysteron-based metamaterials consist of precurved beams encased in rigid 3D printed clamps (size H) and serially coupled to a spring which consists of a vertical beam of thickness $T_{s}$ and one (for a three-hysteron system) or two (for a two-hysteron system) elliptic rings in series with major axis $E_{i}$, minor axis $e_{i}$, and thickness $T_{e}$. At rest, each beam i has a profile $w_{i}=\frac{A_{i}}{2}cos\left(2\pi x/H\right)$, thickness $T_{i}$, and depth b; we use the dimensionless thickness $t_{i}=T_{i}/H$ and amplitude $a_{i}=A_{i}/H$. By varying $t_{i}$, $a_{i}$, and the spring stiffness, we control the mechanical response (*SI Appendix*, Fig. S2).

### 5.2. Fabrication and protocol.

The flexible parts of the samples are two component polyvinyl siloxane rubber (Elite Double 22, Zhermarck) fabricated by standard molding techniques, whereas the rigid parts consist of 3D printed PLA frames (Ultimaker S5; white parts in, e.g., Fig. 1 *B* and *E*). The mechanical response of our samples is probed in a uniaxial testing machine (Instron 5965) which controls the axial displacement U better than 4μm; we use a 100 N sensor, which accurately measures the force down to 0.5 mN. The experiments are performed at 0.05mm/s. We image the sample at a frame rate of 0.41fps, using a CMOS camera (Basler asA2040-25gm/gc).

### 5.3. Specific Designs.

All data (experimental and numerical) presented in the main text are obtained with H=20mm, b=10mm, $t_{1}=0.1$, $a_{1}=0.4$, $T_{e}=2\;$mm, $T_{s}=1.5\;$mm, $E_{1}=E2=7\;$mm and $e_{1}=e_{2}=4.5\;$mm. The two-hysterons samples shown in Fig. 3 have the following parameters: Fig. 3*C*$a_{2}=0.21$, $t_{2}=0.13$; Fig. 3*D*: $a_{2}=0.2$, $t_{2}=0.1$; Fig. 3*E*: $a_{2}=0.2$, $t_{2}=0.11$. Additionally the three-hysterons sample presented in the main text has the following geometrical parameters: $a_{2}=0.24$, $t_{2}=0.11$; $a_{3}=0.2$, $t_{3}=0.12$, $E_{1}=7\;$mm, $e_{1}=4.5$ mm.

### 5.4. Numerical Simulations.

We perform numerical simulation with the commercial software package ABAQUS/EXPLICIT 6.19. In accordance with the experiments, we use Neo-Hookean hyperelasticity with Young’s modulus of 789.49kPa and Poisson’s ratio of 0.49. We mesh the simulation models with four-node plane stress (CPS4R) elements, use a loading speed of 0.05mm/s, and introduce damping to dissipate vibrations caused by snap-through events

### 5.5. T-Graph Construction and Sampling.

We sample the design space by randomly selecting values for $u_{i}^{\pm }$ and $d_{i}$ between zero and one, using the mapping $c_{\mathrm{ij}}=-d_{j}$, and requiring $u_{i}^{-}<u_{i}^{+}$ and $u_{1}^{+}>u_{2}^{+}>\cdots$ which takes care of trivial permutations of the hysterons (20, 21, 24). We use a previously described recursive algorithm to determine the corresponding t-graph (24). In essence, starting from the saturated state {00,⋯}, we determine its up transition and landing state. We then iteratively determine the up and down transitions from all fresh landing states, until no new fresh states can be found. The resulting t-graph contains all N nodes that are reachable from the saturated states and 2N−2 directed edges which represent the up and down transitions. Graphically, we order the nodes from bottom to top as function of their magnetization $m=\Sigma s_{i}$, and from left to right lexicographically. Up and down transitions are colored blue and red. We distinguish between one-step transitions, where a single hysteron flips its phase, and avalanches, which feature multiple hysteron flips, and which are composed by merging several elementary transitions (1, 5, 24, 25). We represent the length of an avalanche, i.e., the number of elementary transitions, by the thickness of the edge—for a one-step transition, the length equals one (see *SI Appendix* for more details).

## Supplementary Material

Appendix 01 (PDF)

Movie S1. Demonstrate the breaking of l-RPM shown in, respectively, Figs. 4a and 4b of the main text.

Movie S2. Demonstrate the breaking of l-RPM shown in, respectively, Figs. 4a and 4b of the main text.

Movie S3. Demonstrate the transient response (τ = 2) shown in, respectively, Figs. 4c and 4d of the main text.

Movie S4. Demonstrate the transient response (τ = 2) shown in, respectively, Figs. 4c and 4d of the main text.

Movie S5. Demonstrate respectively FSM 1, FMS 2 and FMS 3 shown in Figs. 5a-c, 5d-f and 5g-h of the main text.

Movie S6. Demonstrate respectively FSM 1, FMS 2 and FMS 3 shown in Figs. 5a-c, 5d-f and 5g-h of the main text.

Movie S7. Demonstrate respectively FSM 1, FMS 2 and FMS 3 shown in Figs. 5a-c, 5d-f and 5g-h of the main text.

Movie S8. Demonstrates FSM 4 shown in Figs. 6e-f of the main text.

## Data Availability

All study data are included in the article and/or supporting information. Additional Excel files, Python codes, and raw images that support the findings of this study are openly available in Figshare: https://figshare.com/projects/Data_for_Controlled_pathways_and_sequential_information_processing_in_serially_coupled_mechanical_hysterons_/203070 (47).
