# Supplementary material for: Controlled pathways and sequential information processing in serially coupled mechanical hysterons
Source: Proc Natl Acad Sci U S A. 2024 May 20;121(22):e2308414121. doi: 10.1073/pnas.2308414121 (PMC11145188; doi:10.1073/pnas.2308414121)
Supplement: Supplementary file 1 — Appendix 01 (PDF) [file pnas.2308414121.sapp.pdf]

Supplementary Information for

Controlled Pathways and Information Processing in Serially Coupled Mechanical Hysterons.

Jingran Liu, Margot Teunisse, George Korovin, Ivo R. Vermaire, Lishuai Jin, Hadrien Bense, Martin van Hecke

Jingran Liu

This PDF file includes:

Supplementary text
Figs. S1 to S6 (not allowed for Brief Reports)
Table S1 (not allowed for Brief Reports)
Legends for Movies S1 to S8
SI References

Other supplementary materials for this manuscript include the following:

Movies S1 to S8

Supporting Information Text

Supplementary videos. Supplementary videos S1-S8. Front view of a three-hysteron metamaterial described by the graph $3A12$ during stretch-release cycles. The traction speed is 0.05 mm/s, the movies were recorded at a frame rate of 0.41 Hz and accelerated 40 times.
Movies S1 and S2 demonstrate the breaking of l-RPM shown in, respectively, Figs. 4a and 4b of the main text.
Movies S3 and S4 demonstrate the transient response ($\tau = 2$) shown in, respectively, Figs. 4c and 4d of the main text.
Movies S5, S6 and S7 demonstrate respectively FSM 1, FMS 2 and FMS 3 shown in Figs. 5a-c, 5d-f and 5g-h of the main text.
Movies S8 demonstrates FSM 4 shown in Figs. 6e-f of the main text.

Model of hysterons in series. We consider n serially coupled hysterons under quasistatic, overdamped dynamics. The force-displacement relation of hysterons is modeled by a bilinear relation:

$$f_j = (u_j - d_j s_j) \ , \tag{1}$$

with f_j the force, u_j the displacement, d_j the force discontinuity and s_j the state. Sweeps of the global deformation U yields smooth episodes interspersed with discontinuous transitions. In each smooth episodes, $\{s_j\}$ is constant, and mechanical equilibrium yields relations between the global and local deformations:

$$U = \sum_j u_j \ . \tag{2}$$

$$F = f_i = f_j \text{ for all } i, j, \tag{3}$$

The force balance equations can be used to link the extensions of pairs of hysterons as $u_j = (u_i - d_i s_i) + d_j s_j$. By systematically eliminating all u_j for $j \neq i$, we can then express, for fixed s_j, the global deformation U as a function of any single u_i, and vice-versa,

$$U = n(u_i - d_i s_i) + \sum_j d_j s_j \ , \tag{4}$$

$$u_i = d_i s_i + \frac{1}{n}(U - \sum_j d_j s_j) \ . \tag{5}$$

Transitions. Consider sweeping U so that hysteron i flips its internal state; due to the mechanical balance equations, the force and the deformations of all hysterons change. To calculate these changes, we note that just before this transition u_i approaches one of the local switching fields $u_i^\pm$, and that the global driving U just before and just after a transition are equal. Using Eq. (4), we find:

$$U_i^\pm(S) = n(u_i^\pm - d_i s_i) + \sum_j d_j s_j. \tag{6}$$

Denoting the states and deformations after the flipping event as $\{s'\}$ and $\{u'_j\}$, making use of the fact that $s'_j = s_j$ if $j \neq i$, and using Eq. (5), we find the following explicit expressions for the discontinuous changes in the hysteron deformations and forces:

$$\text{hysteron } i\text{:} \quad u'_i - u_i = (1 - \frac{1}{n}) d_i (s'_i - s_i) \ , \tag{7}$$

$$\text{hysteron } j \neq i\text{:} \quad u'_j - u_j = -\frac{d_i}{n}(s'_i - s_i) \ , \tag{8}$$

$$\text{all hysterons } k\text{:} \quad f'_k - f_k = -\frac{d_i}{n}(s'_i - s_i) \ . \tag{9}$$

We note that these expressions take the correct form for $n = 1$, that all forces jump by the same amount as required by force balance, that a $0 \to 1$ ($1 \to 0$ transition) lowers (increases) the force, and that the sum of all deformations adds up to zero as required by length conservation (Eq. 4). We note that as the relation between U and u_i depends on the state of all hysterons (Eq. 4,5), the expressions for the changes in u_i are cumbersome.

Mapping serial hysterons to explicit model. To map the system of n serially coupled hysterons (Eqs. 1-3) to an explicit model of interacting hysterons (1) , we focus on mapping the expression for U_i^c for serial hysterons (Eq. 6) to the expression for $V_i^\pm$ for interacting hysterons:

$$V_i^\pm(S) = v_i^\pm - \sum_j c_{ij} s_j \ . \tag{10}$$

Importantly, the explicit model allows to gauge out all diagonal terms in c_{ij} ('self-coupling') by modifying the lower bare switching fields v_i^-, and also allows for a rescaling of c_{ij}, provided the gaps between the upper and lower bare switching fields are scaled accordingly (1). Hence, there is no unique mapping from the serial to the explicit model; for completeness we give three mappings here. In all cases, we only require that $v_i^+ > v_i^-$, which is a weaker requirement than $u_i^+ > u_i^-$.

Mapping I: This mapping simply defines

$$V_i^{\pm} = U_i^{\pm} , \quad [11]$$
$$v_i^{\pm} = n(u_i^{\pm} - d_i s_i) , \quad [12]$$
$$c_{ij} = -d_j . \quad [13]$$

Note that the upper bare switching field can also be simplified as $v_i^+ = n u_i^+$, as $s_i = 0$ for an up transition of hysteron i. Substituting these definitions in Eq. (10) yields the desired form $U_i^{\pm} = u_i^{\pm} - \Sigma_j c_{ij} s_j$. Notice that in this mapping, $c_{ii} \neq 0$ and the bare upper and lower switching fields scale with n.

Mapping II: To gauge out the self-coupling term, we define

$$V_i^{\pm} = U_i^{\pm} , \quad [14]$$
$$v_i^{+} = n u_i^{+} , \quad [15]$$
$$v_i^{-} = n(u_i^{-} - d_i) + d_i , \quad [16]$$
$$c_{ij} = -d_j \text{ for } i \neq j , \quad [17]$$
$$c_{ii} = 0 . \quad [18]$$

For the upper global switching field we find $U_i^+(S) = n u_i^+ + \Sigma_{j\neq i} d_j s_j$. As $s_i = 0$, we can rewrite the first term as $n(u_i^+ - d_i s_i)$, and the second term as $\Sigma_j d_j s_j$, thus obtaining the desired form Eq. (6) for upper global switching field $U_i^+(S)$. For the lower global switching field we find $U_i^-(S) = n(u_i^- - d_i s_i) + d_i + \Sigma_{j\neq i} d_j s_j$. As $s_i = 1$ for a down transition, we can rewrite the last two terms as $\Sigma_j d_j s_j$, thus obtaining the desired form Eq. (6) for lower global switching field $U_i^-(S)$.

Mapping III: To gauge out the self coupling term and to have the bare switching fields of both models be similar, we define

$$V_i^{\pm} = U_i^{\pm}/n , \quad [19]$$
$$v_i^{+} = u_i^{+} , \quad [20]$$
$$v_i^{-} = (u_i^{-} - d_i) + d_i/n , \quad [21]$$
$$c_{ij} = -d_j/n \text{ for } i \neq j , \quad [22]$$
$$c_{ii} = 0 , \quad [23]$$

which by substitution yields the desired form Eq. (6). In this mapping, instead of the extensive quantity 'total deformation', we use the intensive strain-like quantity V_i, which is the deformation per hysteron. The upper bare switching fields in serial and explicit model are equal and system size independent. Our mapping also clarifies that the interactions come from a global coupling, and vanish in the limit of large n. Finally, the lower switching field v_i^- is a bit more complex. It contains both a system size independent shift $(-d_i)$ and a system size dependent shift $(+d_i/n)$ with respect to the bare switching fields u_i^- — please note that the size dependent shift is due to the convention that $c_{ii} = 0$.

T-graph construction. An important restriction for our t-graphs is that we require each elementary transition to only have one unstable hysteron (1). However, for some parameter values this requirement is violated. Consider hysteron i in state S becoming unstable at a certain driving value U^c. This defines then a landing state S'. Due to interactions, it can not be ruled out that hysterons are unstable in state S' at $U = U^c$. If one hysteron is unstable, this leads to an avalanche. However, if more than one hysteron is unstable (say hysteron l and k), the situation is more complex and features a race condition: flipping first hysteron l and then k may lead to a different scenario than first flipping k and then l — hysteron flips do not commute. Alternatively, one may flip both hysterons simultaneously, which may lead to a different situation again. More work is needed to understand the physical reality of such situations, and whether a simple coupled hysteron model can consistently describe such situations. Hence, while these type of transitions may enlarge the range of t-graphs, in our studies here we have excluded these transitions and label the system then ill-defined (1).

Avalanches. In general, avalanches refer to transitions involving multiple hysteron flips; they are referred to as 'up' or 'down' depending on whether triggering them requires the increase or decrease of the global driving, and their length is defined by the number of elementary transitions separating the initial and final states (1). As an example of an avalanche, we consider graphs $2A$ and $2A1$ (Fig. 2a main text). State $\{01\}$ has an up transition to $\{11\}$ at $U^+(\{01\})$, while $\{11\}$ has a down transition to $\{10\}$ at $U^-(\{11\})$. Hence, when the system is in state $\{01\}$ and U is increased beyond the critical driving $U^c = U^+(\{01\})$, there is an initial transition to state $\{11\}$. If $\{11\}$ is stable at $U = U^c$, the transition terminates, and the system features a one-step transition $\{01\} \to \{11\}$ (see graph $2A$). However, if $U^-(\{11\}) > U^+(\{01\})$, S_1 is unstable at U^c and immediately transitions to $\{10\}$ (1–3). The two elementary transitions $\{01\} \to \{11\}$ and $\{11\} \to \{10\}$ then merge and $\{11\}$ is an experimentally unobservable intermediate state. Assuming $\{10\}$ to be stable at $U = U^C$, the result is a single 'up' avalanche transition of length two: $\{01\} \xrightarrow{up} \{10\}$ (see graphs $2A1$).

Scrambling. A scrambled t-graph features pairs of transitions that are not consistent with a global ordering of the switching fields. As an example of state dependent ordering of the switching fields, consider the pair of transitions $\{000\} \to \{001\}$ and $\{100\} \to \{110\}$. The presence of the first transition indicates that in state $\{000\}$ the up switching field for the third hysteron is lower than the up switching fields of the other hysterons, i.e., $u_3^+(000) < u_2^+(000)$ and $u_3^+(000) < u_1^+(000)$; while the second transition indicates that in state $\{100\}$, the up switching field of the second hysteron is lower than the up switching field of the third hysteron, i.e., $u_3^+(100) > u_2^+(100)$. Such pairs of scrambled transitions cannot be observed in the absence of interactions, as in that case the upswitching fields are state independent ($u_j^+(S) = u_j^+(000)$), but are an important contributor to the wide variety of t-graphs observed for general interactions. We note that due to the specific nature of their coupling, scrambling is not possible in systems of serial hysterons (see section below).

General properties of simple SC-graphs. To discuss general features of the t-graphs for serially coupled hysterons, we use the mapping to the explicit model of interacting hysterons. While coupled hysterons with arbitrary interactions feature a very wide palette of t-graphs (1, 2), for the specific case of serially coupled hysterons the t-graphs are restricted.

First, simple SC-graphs do not feature scrambling, so that the sequence of hysteron switches on the main loop completely determines the elementary transitions in the rest of the graph. This follows directly from the definition of the global antiferromagnetic interactions $c_{ij} = -d_j$. For a given state S, the effective switching fields can be written as $U_i^\pm = u_i^\pm - c_{ij}s_j = u_i^\pm + d_j s_j$ (using the mapping to the explicit model). Hence, the shift of the switching fields for a given state is the same for each hysteron i, the ordering of $U_i^\pm$ and $u_i^\pm$ are the same, and the interactions do not impact the switching hysteron. Hence, a simple SC-graph without avalanches is a P-graph.

Second, all avalanches are of length two, are obtained by merging an up and a down transition and thus connect two states with the same magnetization $m := \Sigma_i s_i$, and for a given monotonic sweep of U, no two avalanches can occur in succession. To understand this, we note that avalanches must be composed of alternating up and down (elementary) transitions when the interactions are purely antiferromagnetic (1, 4); hence during such an avalanche the magnetization $M(S)$ must alternatingly increase and decrease by one. Moreover, all simple sc-graphs can be constructed by starting from avalanche-free and scrambling-free t-graphs—they are precisely the Preisach graphs—and then merging pairs of elementary transitions to obtain length two avalanches. The Preisach graphs satisfy l-RPM, which restrains the structure of transitions within a loop. Suppose now that one has a series of elementary transitions $S^{(0)} \to S^{(1)} \to S^{(2)} \to S^{(3)}$ which form an avalanche. Let the first transition ($S^{(0)} \to S^{(1)}$) be an up transition (the same argument can be constructed if it is a down transition). Then, the second ($S^{(1)} \to S^{(2)}$) must be a down transition, so that $S^{(1)}$ must be the endpoint of a loop of which $S^{(0)}$ and $S^{(2)}$ lie on the up and down boundaries, respectively. Due to l-RPM, the up transition $S^{(2)} \to S^{(3)}$ cannot lead to any other state than $S^{(1)}$. To see this, consider the up-orbit from $S^{(2)}$, which under l-RPM must go through the endpoint $S^{(1)}$. However, since $S^{(2)} \to S^{(3)}$ is an up transition, $M(S^{(3)}) = M(S^{(1)})$ and subsequent up transitions can never lead back to the state $S^{(1)}$. Thus, the up transition $S^{(2)} \to S^{(3)}$ is only allowed under l-RPM if $S^{(3)} = S^{(1)}$. An avalanche cannot have any repeating nodes, as this would lead to a self-loop. Therefore, one cannot have avalanches of $l = 3$ and higher when there are only anti-ferromagnetic interactions and the base graphs obey l-RPM.

The argument above can be extended to show that no up avalanche can start from the final state of another up avalanche (similar for down avalanches). To see this, we consider an up avalanche composed from the elementary transitions $S^{(0)} \xrightarrow{up} S^{(1)} \xrightarrow{down} S^{(2)}$. As we showed before, $S^{(2)} \xrightarrow{up} S^{(1)}$, and since $S^{(1)} \xrightarrow{down} S^{(2)}$ the chain of states is $S^{(0)} \xrightarrow{up} S^{(1)} \xrightarrow{down} S^{(2)} \xrightarrow{up} S^{(1)} \xrightarrow{down} S^{(2)}$. The first two transitions can merge under an avalanche— $S^{(0)} \xrightarrow{up,ava} S^{(2)}$, but the latter two transitions would form a loop, which is forbidden

Return Point Memory and l-RPM. Return point memory (RPM) is the widespread ability of complex systems to 'remember their extremal driving', i.e., to return to a previously visited state when the driving revisits an extremum value (5–8). Anti-ferromagnetic interactions can break RPM (4). The presence of strict RPM cannot be determined from the topology of a t-graph as it may depend on the precise values of the switching fields (9). We therefore use a recent definition of so-called loop-RPM (l-RPM) which can be determined from the topology of the t-graph (10, 11). l-RPM requires that all loops within a t-graph are absorbing. In essence, a loop is defined by a pair of nodes S_m and S_M, where the system evolves from S_m to S_M and vice-versa by a series of up and down transitions. The intermediate states are defined as the up (down) boundaries of this loop. l-RPM then requires states S_m (S_M) to be accessible by a sequence of down (up) transitions from any up (down) boundary state. Informally, l-RPM requires that any orbit starting from a state in a given loop escapes this loop by going through either the top or bottom state. We finally note that broken l-RPM implies broken RPM, that breaking l-RPM necessitates either scrambling or avalanches, and that t-graphs with broken l-RPM allow for more complex behavior than t-graphs that satisfy l-RPM (1).

Full hierarchy of t-graphs. We present in Fig. S1 the 44 simple sc-graphs found for $n = 3$ serially coupled hysterons hierarchically organized following the rules exposed above and in the main text. Our numerical sampling is exhaustive and we obtain precisely 44 simple SC-graphs, with six P-graphs ($A - F$), and respectively 12, 11, 8, 4, 2 and 1 simple sc-graphs with 1, 2, 3, 4, 5 and 6 avalanches. As explained above, all avalanches connect two states with the same magnetization $m := \Sigma_i s_i$ and are of length two.

To understand how these 44 simple sc-graphs emerge, we organize them hierarchically. We start from the six Preisach graphs ($3A$-$3F$), and consider all possible length-two avalanches obtained by merging transition pairs. Collecting all possible

avalanches, we find that for for $m = 2$, all six potential avalanches that satisfy these conditions occur. For $m = 1$, only three of these occur, with three others forbidden due to the ordering of the upper switching fields. Specifically, $\{100\} \xrightarrow{down} \{010\}$ is forbidden as its intermediate state, $\{000\}$ must transition to $\{001\}$, not $\{010\}$; $\{001\} \xrightarrow{up} \{100\}$ is forbidden as its intermediate state, $\{011\}$ cannot reach $\{100\}$ in one elementary transition; and similarly, $\{010\} \xrightarrow{up} \{100\}$ is forbidden as its intermediate state, $\{011\}$ cannot reach $\{100\}$ in one elementary transition.

For a given Preisach graph, not all possible avalanches can occur simultaneously— i.e., no graph can have $\{011\} \xrightarrow{up} \{101\}$ and $\{011\} \xrightarrow{up} \{110\}$, and which of these two is allowed is set by the down transition from state $\{111\}$. After such considerations, we can determine the potential avalanches for each 'base' graph ($3A$-$3F$) and obtain the remaining 38 simple sc-graphs by combining these, leading to a natural hierarchy (Fig. S1).

Thus combining avalanches, we find a total of 45 simple sc-graphs. However, there is no guarantee that all combinations are realizable, and indeed we find one example of a putative graph—$3B23$— that cannot be realized. We now ask why this graph is not realizable. A graph is realizable if the underlying set of design inequalities relating the switching fields has a solution. We reason that all four transitions $\{001\} \xrightarrow{up} \{011\}$, $\{011\} \xrightarrow{up} \{111\} \xrightarrow{down} \{110\}$, $\{110\} \xrightarrow{down} \{010\}$ and $\{010\} \xrightarrow{down} \{000\} \xrightarrow{up} \{001\}$ must be involved in the combination of inequalities that is incompatible, since more than one of the candidate graphs would otherwise be impossible.

To know whether a set of inequalities is solvable, one can use the Fourier-Motzkin method for elimination of variables. In the Fourier-Motzkin method, a variable x is eliminated by combining the inequalities to create a new set that does not contain x. A set of inequalities does not have a solution if, after all variables are eliminated, a contradictory statement remains such as $0 > 0$. By working backwards, we can identify which combinations of inequalities lead to a contradictory statement and are therefore incompatible.

We find that for the putative graph $3B23$, there is a single combination of six inequalities that is incompatible, namely:

$$\begin{aligned} u_1^- - c_2 &> u_2^- - c_1 \\ u_3^+ &> u_1^- \\ u_2^- &\geq u_3^+ \\ u_2^+ - c_3 &> u_3^- - c_2 \\ u_1^+ &> u_2^+ \\ u_3^- - c_1 &\geq u_1^+ - c_3 \end{aligned} \qquad [24]$$

We further note that the six inequalities arise from the four transitions$\{001\} \xrightarrow{up} \{011\}$, $\{011\} \xrightarrow{up} \{111\} \xrightarrow{down} \{110\}$, $\{110\} \xrightarrow{down} \{010\}$ and $\{010\} \xrightarrow{down} \{000\} \xrightarrow{up} \{001\}$, in agreement with our prediction. Graph $3B23$ thus illustrates that it is not trivial to see whether a combination of transitions of compatible, and that it therefore remains necessary to explicitly check whether candidate graphs are solvable by generating the design inequalities.

Experimental realizations. The geometrical parameters of the different elements constituting a hysteron play a crucial and complex role on its mechanical response, and thus on the subsequent pathways. We used our numerical simulations to investigate the mechanical response of a single hysteron. The stiffness of the spring governs the hysteretic behavior of the beam, and we find that for a given beam design, the spring has to be soft enough for the system to be bistable (Fig. S2b). The stiffness of the spring, however, does not influence the force threshold. Fig. S2c illustrates how the force threshold, as defined in Fig. S2b evolves with the geometry of the pre-curved beam.

We fabricated different samples described by the simple sc-graph $3A12$, which we denote A,B,C,D. Their geometrical properties are:

Sample A: $a_1 = 0.4$, $t_1 = 0.1$, $a_2 = 0.24$, $t_2 = 0.11$, $a_3 = 0.2$, $t_3 = 0.12$, $E1 = 7$mm, $E2 = 4.5$mm;
Sample B: $a_1 = 0.4$, $t_1 = 0.1$, $a_2 = 0.24$, $t_2 = 0.11$, $a_3 = 0.2$, $t_3 = 0.12$, $E1 = 8$mm, $E2 = 4.5$mm;
Sample C: $a_1 = 0.4$, $t_1 = 0.1$, $a_2 = 0.23$, $t_2 = 0.11$, $a_3 = 0.2$, $t_3 = 0.12$, $E1 = 7$mm, $E2 = 4.5$mm;
Sample D: $a_1 = 0.4$, $t_1 = 0.1$, $a_2 = 0.23$, $t_2 = 0.11$, $a_3 = 0.2$, $t_3 = 0.12$, $E1 = 8$mm, $E2 = 4.5$mm;
where $L = 20$ mm, $b = 1$ mm. We provide in Fig. S3 the t-graph for sample A with all the switching fields.

Fig. S4 presents the full experimental response of sample A subjected to driving protocol 'yxxxyyxy' described in the main text (see Fig. 6 of the main text). Each state transition is denoted by a vertical grey line, and the state reached is indicated above the plot.

Transients. A popular characterization of the transient is the number of driving cycles τ before the system falls into a periodic orbit of period T (1, 9). To connect the transient to i_1—the index of the first repeating state—we define $t_1 := i_1/2$; note that t_1 can take on half-integer values, and τ can be obtained by rounding up t_1 to the nearest integer. Similarly, rounding up $(i_2 - i_1)/2$ yields the integer period T. For collections of $n = 3$ non-interacting hysterons, we found that (i_1, i_2) can only take on four values $(0, 1), (0, 2), (1, 2)$ and $(1, 3)$, which follows from the properties of strict RPM, and this remains true in the limit of large n. In contrast, sample A can feature longer transients: (i_1, i_2) can take on values $(2, 3), (2, 4), (3, 4)$ (the latter corresponding to the transient $\tau = 2$ exhibited in Fig. 4c,d of the main text) in addition to the four values listed above

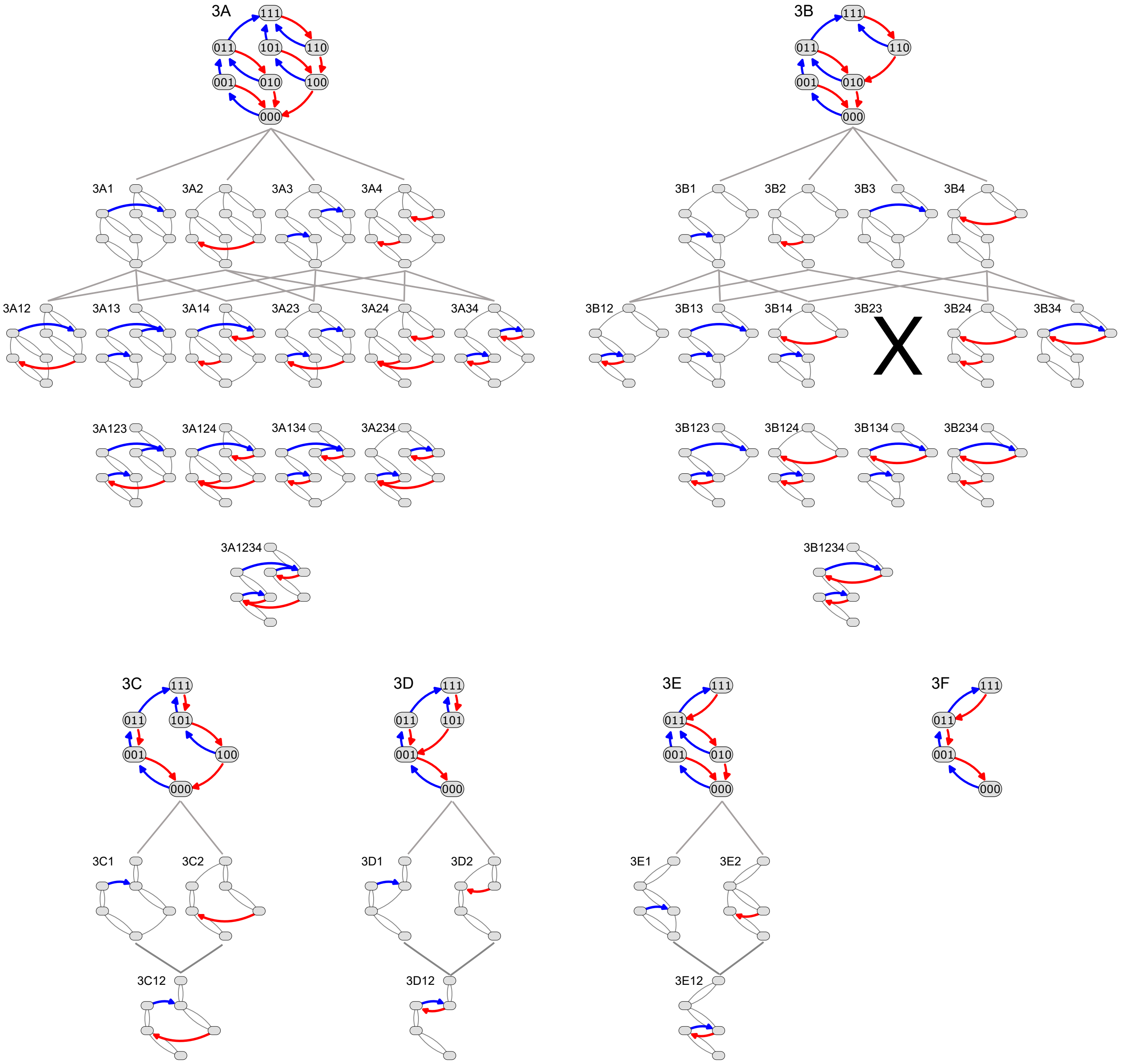

Fig. S1. 44 tgraphs. The P-graphs $3A$-$3F$ have a varying number of daughter graphs. In each daughter graph, we use a colored arrow to indicate the avalanches that replace the direct transitions in the mother graphs — the remaining direct transitions are as in the mother graph and represented in grey and without arrowheads. $3A$ has four groups of avalanches (1-4) that occur in any combination. Note that the individual avalanches in group 3 and 4 cannot occur independently. $3B$ has four avalanches (1-4) that can occur in almost any combination - with the exception of t-graph $3B23$ which cannot be realized with serial coupling. Notice that avalanches 2 and 3 can occur together when either avalanche 1 or 4 is present. $3C$, $3D$ and $3E$ all allow two avalanches that can occur together. $3F$ has no daughter graphs, because all its states have different values of m. Grey lines indicate daugther relations in graphs $3C$, $3D$ and $3E$ and in the first two levels of $3A$ and $3B$.

(Fig. S5). We note that we can use the plots of i_1 and i_2 to calculate statistical measures. For example, to calculate the probability of finding a certain transient (e.g., $i_2 = 2$) for randomly sampled values of U^m and U^M, one can add the volumes in U^p, U^q space for each matching block (Fig. S5).

We additionally have studied (i_1, i_2) for large collections of randomly sampled simple SC-graphs, for $n = 3, 4$ and 5. For $n = 3$, we find the same values for (i_1, i_2) as for sample A. For larger n, longer but statistically rare transients can be found, and $i_2 - i_1 \leq 2$, since there are no subharmonic orbits (Fig. S5c). We finally note that for general interactions for $n = 3$ we both observed longer transients and subharmonic orbits, with (i_1, i_2) taking on values such as $(4, 6), (0, 4)$ and $(0, 6)$ (1).

Determining classes of FSMs. To determine all FSMs for a given t-graph and alphabet we use a simple recursive algorithm. We determine all states $\{S_0\}$ that are stable at $U = U^p$, and build candidate FSMs by iteratively determining $a(S_0)$, $b(S_0)$,

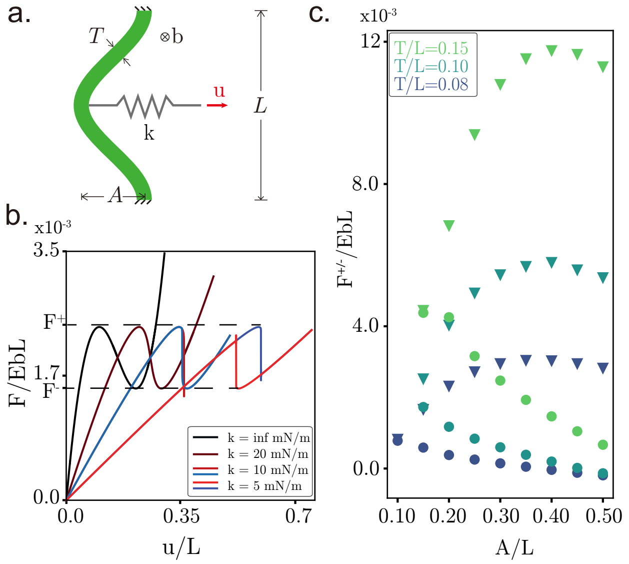

Fig. S2. Properties of a single hysteron (a) Schematic of a single mechanical hysteron, represented by a precurved beam of Young's modulus E, in series with a spring. (b) Numerical results. Normalized force as a function of the normalized displacement of a single hysteron of fixed geometry for different values of the spring stiffness k. We note that bistability appears for $k < 10$ mN/m. $L = 20$ mm, $b = 1$ mm, $a = 0.15$, $t = 0.1$. (c) Numerical results. Normalized threshold force (as defined in Fig. S2b) as a function of the normalized amplitude of a single hysteron in series with an infinitely rigid spring for different thicknesses. ○: F^-, ▽: F^+.

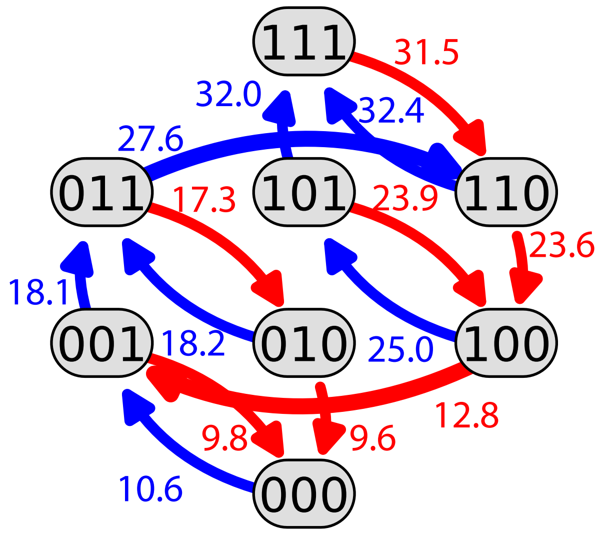

Fig. S3. Full t-graph of sample A Sample A is described by t-graph $3A12$, next to each arrow is indicated the related critical switching field in mm.

$a(a(S_0))$ etc (where $a, b, \ldots$ are characters in the alphabet), until no new states are found, for each S_0. For the specific case of two pulses driving, this yields transition tables as function of p, q_a, q_b and S_0. To turn these into candidate FSMs, we take S_0 as initial state, label it grey, and select one or more accepting states — we do not consider each of these latter choices separately. Many of the candidate FSMs thus obtained are equivalent under permutations of the characters $a \leftrightarrow b$ or permutations of the (non-initial) states, and such permutations do not alter the computational complexity. We therefore determine the irreducible FSMs which are distinct under such permutations.

We finally note that there are two classes of such graphs: each either has one or no states without incoming edges. If there is one such state, it must be S_0 (all the other nodes can be reached starting from S_0, so they have incoming edges). However, some graphs have no nodes without incoming edges. In that case, all nodes can be chosen as initial nodes - the candidate graphs contain graphs with each of these nodes as initial node. To avoid double counting, we represent such a group of graphs with a single graph without a (grey) labeled initial state - in such cases, each state can act as initial state (See Fig. 6d of the main text).

Sample	A	B	C	D
$U^{+}(000)$ [mm]	10.5999	11.40003	10.31042	12.3449
$U^{-}(001)$ [mm]	9.84076	10.93345	9.9276	11.2709
$U^{+}(001)$ [mm]	18.09459	19.18761	17.32536	18.95538
$U^{-}(010)$ [mm]	9.59519	10.39483	9.61796	10.68024
$U^{+}(010)$ [mm]	18.15037	19.06499	17.67501	19.23055
$U^{-}(100)$ [mm]	12.84579	14.07818	13.21251	14.15104
$U^{+}(100)$ [mm]	25.02025	25.79533	24.37527	26.57042
$U^{-}(011)$ [mm]	17.26535	18.42977	17.21478	18.07036
$U^{+}(011)$ [mm]	27.61595	29.12643	27.05683	28.88672
$U^{-}(101)$ [mm]	23.94463	25.11994	23.88999	25.25541
$U^{+}(101)$ [mm]	31.95013	33.25018	31.04495	32.60016
$U^{-}(110)$ [mm]	23.63954	24.62019	23.43203	24.62002
$U^{+}(110)$ [mm]	32.44143	33.43623	31.68213	33.37654
$U^{-}(111)$ [mm]	31.50437	32.6992	31.25019	32.25539

Table S1. Switching fields for four different three-hysterons samples described by t-graph $3A12$.

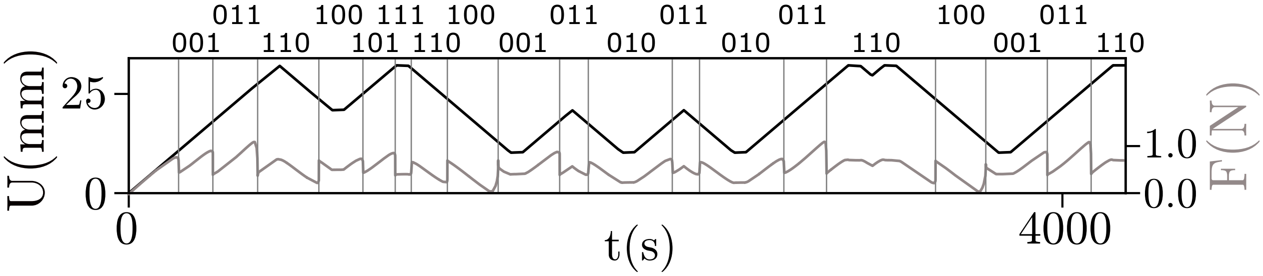

Fig. S4. Experimental response of sample A to driving protocol 'yxxxyyxy'. Global force F (grey line) and deformation U (black line) as a function of time corresponding to the full FSM shown in Fig.6 of the main text. Each state reached is labeled on top of the plot.

FSMs for non-interacting hysterons. To understand the emergence of the limited set of eight FSMs for Preisach systems, we note that for independent hysterons, positive or negative pulses only affect individual hysterons. Hence, the operators a_p^{q1} and a_p^{q2} now act on individual hysterons, so that $a_p^{q1}(s_i)$ is well defined.

We first consider the case where the operators a_p^{q1} and a_p^{q2} represent positive pulses (the case of two negative pulses follows by symmetry). We then order these two pulses such that $|q_2 - p| > |q_1 - p|$; in other words, a_p^{q1} represents a weak pulse and a_p^{q2} a strong pulse. We can distinguish three groups of hysterons, by how they transform their phase s_i under action of a_p^{q1} and a_p^{q2}. Group one contains the hysterons that do not change under the action of either pulses ($a_p^{q1}(s_i) = s_i$ and $a_p^{q2}(s_i) = s_i$); group two contains the hysterons that are set to one by the strong pulse but not by the weak pulse ($a_p^{q1}(s_i) = s_i$ and $a_p^{q2}(s_i) = 1$); group three contains the hysterons that are set to one by either pulse ($a_p^{q1}(s_i) = 1$ and $a_p^{q2}(s_i) = 1$).

By considering different combinations of groups of hysterons (i.e., only group one, group one and group two, etc), we find four distinct FSMs. Labeling the eight FSMs in Fig. 6d of the main text that arise for no interactions as FSM 1 - FSM 8 (top to bottom), these correspond to FSM 1 - FSM 4 (where weak and strong pulses correspond to yellow and green transitions respectively). These four FSMs are the only ones possible for pulses of equal sign, as can also be understood from strict RPM.

When $a_p^{q1}(s_i)$ and $a_p^{q2}(s_i)$ represent positive and negative pulses, the situation is slightly more complex. We assume that $q_1 > p$ and $q_2 < p$ (the opposite case follows by symmetry). Each hysteron can now either be set to 1 by the action of a_p^{q1} or left invariant; and each hysteron can now either be reset to 0 by the action of a_p^{q2} or left invariant. Together, this yields four groups of hysterons. Again considering all permutations yields the remaining FSM types 5-8, as well as FSM 1-4 (Fig. 6d). As we will discuss elsewhere, there are two subtleties for pulses of opposite sign: First, if one includes so-called Garden of Eden states, there is a nineth FSM that can be created; second, one can get additional FSMs when one considers three distinct pulses.

From FSMs to t-graphs. Here we show how we can realize any FSM by a t-graph. Consider an FSM with N states and L characters, and a transition table $a_i(S_j) = S_k$ (Fig. S6a). First, we encode the alphabet $\{a_i\}$ for $i = 1, \cdots$ as pulses a_0^i, so that an input string such as $'a_3 a_1 a_5'$ is translated to a sequence of driving pulses maxing out at $U = 3, 1$ and 5, respectively. For convenience, we split the driving pulses in positive ramps, i.e., use $a_0^i(S_j) = r_i^0(r_0^i(S_j))$ ((Fig. S6b).). Second, we define $N \times L$ states S_j^i, such that S_j^0 are all stable at $U = 0$ — these correspond to the N states S_J of the FSM — and all other states are intermediate states reached during the upsweep of each pulse: $r_0^i(S_j^0) = S_j^i$. The transition table $a_i(S_j) = S_k$ can be realized by a t-graph where a down sweep from $U = i$ to $U = 0$ leads to a transition from S_j^i to S_k^0, in other words, $r_i^0(S_j^i) = S_k^0$ (Fig. S6c).

We finally give an example of how such t-graphs can be realized with interacting hysterons. We note that our construction requires a large number of hysterons, and for most FSMs, more effective implementations can likely be found. Moreover, our construction is for a specific FSM with $N = 3$ states and a single ($L = 1$) character denoted a_1; while it can easily be extended to N states, we have not yet extended this construction to more characters. Although very technical, this construction is a

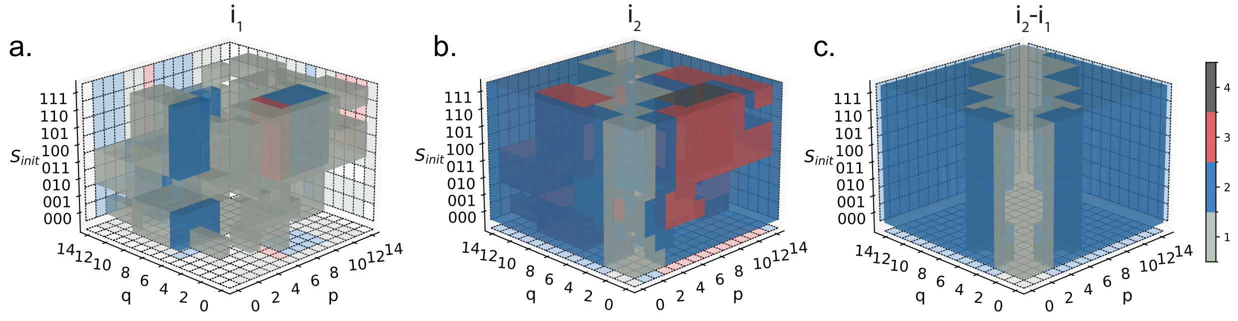

Fig. S5. Transients and periodicity of sample A. (a) Transient duration i_1 as function of initial state and driving amplitudes U^p and U^q (see main text). (b) i_2. (c) Periodicity $i_2 - i_1$.

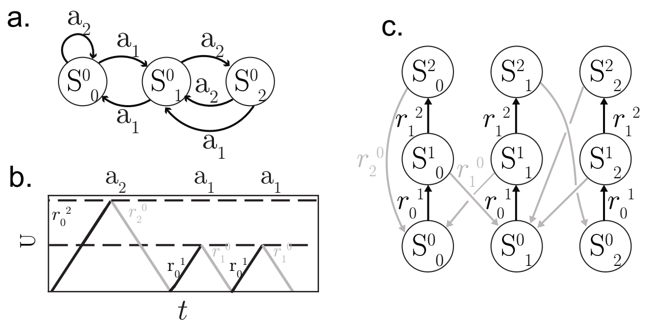

Fig. S6. Link from FSM to t-graph. (a) Example of a 3 states and 2 characters FSM. (b) The alphabet a_i attached to the FSM is decomposed into driving ramps r. (c) Subsequent t-graph obtained by following the described procedure.

starting point for a general construction and we include it here for completeness. The specific transition table we realize follows the example in (Fig. S6c) restricted to the single character a_1: $a_1(S_0) = S_1$; $a_1(S_1) = S_0$; $a_1(S_2) = S_1$. We represent the $N \times L$ states of the t-graph by $N \times (L+1)$ hysterons: First, we encode the 'ground state' S_0^0 and its 'excited states' S_0^j as $\{1000\dots\}$, $\{1100\dots\}$, $\dots$, thus requiring $L+1$ hysterons for each state; similarly, we encode the states S_1^0 as $\{0\dots01000\dots\}$, $\{0\dots01100\dots\}$, where there are $L+1$ leading zeros. For the specific example of $N = 3$ and $L = 1$, the ground states are $\{100000\}$, $\{001000\}$ and $\{000010\}$, and their respective excited states $\{110000\}$, $\{001100\}$ and $\{000011\}$. We define the character 'a_1' as a pulse starting at driving field $U = 0$ and with peak value $U = 1$.

Irrespective of the specific FSM we wish to implement, we take for the switching fields: $u_1^+ = u_3^+ = u_5^+ = 1.5$, $u_2^+ = u_4^+ = u_6^+ = 0.85$, $u_1^- = u_3^- = u_5^- = -0.5$ and $u_2^- = u_4^- = u_6^- = 0.25$ - to avoid degeneracies, we can add a small, fixed random perturbation to each of the bare switching fields. We label hysterons from left to right from 1 to 6, and fix the following interactions coefficients at -0.25, irrespective of the transition table: $c_{4,1}, c_{6,1}, c_{2,3}, c_{6,3}, c_{2,5}, c_{4,5}$. These interactions make sure that any ground state suppresses the switching on of any of the hysterons that encode excitement of another state. We also a second set of interactions coefficients at -0.25 to prevent avalanches to occur: $c_{3,1}, c_{5,1}, c_{1,3}, c_{5,3}, c_{1,5}, c_{3,5}$. To implement each specific FSM transition, we need to implement an appropriate pathway from the excited states S_i^1 to the appropriate ground states S_k^0. Without any additional interactions, we have implemented the trivial FSM where $a_1(S_i) = S_i$. Now, for each i where $a_1(S_i) = S_k$ and $k \neq i$, we add some additional interaction coefficients. In our example, to implement $a_1(S_0) = S_1$, we take $c_{1,2} = -1.3$ and $c_{3,2} = 0.75$. Starting out at the excited state $S_0^1 = \{110000\}$, and decreasing U from 1 to 0, we then first find an avalanche transition to an intermediate state $\{011000\}$, which in turns transitions to the ground state $S_1^0 = \{001000\}$. If we were to require that $a_1(S_0) = S_2$, we would instead take $c_{1,2} = -1.3$ and $c_{5,2} = 0.75$, producing an intermediate state $\{010010\}$ that transitions to the ground state $S_2^0 = \{000010\}$. Hence, by a judicious choice of interaction coefficients, any transition map can be implemented. The specific example $a_1(S_0) = S_1$; $a_1(S_1) = S_0$; $a_1(S_2) = S_1$ requires: $c_{1,2} = c_{4,3} = c_{5,6} = -1.3$ and $c_{2,3} = c_{1,4} = c_{3,6} = 0.75$. We finally note that the corresponding FSM has no self loops, in contrast to all FSMs derived for serially coupled hysterons.

Movie S1. Orbit that breaks l-RPM, Fig. 4a

Movie S2. Orbit that breaks l-RPM, Fig. 4b

Movie S3. Transient response ($\tau = 2$), Fig. 4c

Movie S4. Transient response ($\tau = 2$), Fig. 4d

Movie S5. FSM1, Fig. 5a-c

Movie S6. FSM2, Fig. 5d-f

Movie S7. FSM3, Fig. 5g-h

Movie S8. FSM4, Fig. 6e-f

References

1. M van Hecke, Profusion of transition pathways for interacting hysterons. *Phys. Rev. E* **104**, 054608 (2021).
2. CW Lindeman, SR Nagel, Multiple memory formation in glassy landscapes. *Sci. Adv.* **7**, eabg7133 (2021).
3. NC Keim, JD Paulsen, Multiperiodic orbits from interacting soft spots in cyclically sheared amorphous solids. *Sci. Adv.* **7**, eabg7685 (2021).
4. JP Sethna, et al., Hysteresis and hierarchies: Dynamics of disorder-driven first-order phase transformations. *Phys. Rev. Lett.* **70**, 3347–3350 (1993).
5. JA Barker, DE Schreiber, BG Huth, DH Everett, Magnetic hysteresis and minor loops: models and experiments. *Proc. Royal Soc. London. A. Math. Phys. Sci.* **386**, 251–261 (1983).
6. DH Everett, WI Whitton, A general approach to hysteresis. *Trans. Faraday Soc.* **48**, 749–757 (1952).
7. JM Deutsch, A Dhar, O Narayan, Return to return point memory. *Phys. Rev. Lett.* **92**, 227203 (2004).
8. O Hovorka, G Friedman, Onset of reptations and critical hysteretic behavior in disordered systems. *J. Magn. Magn. Mater.* **290-291**, 449–455 (2005) Proceedings of the Joint European Magnetic Symposia (JEMS' 04).
9. H Bense, M van Hecke, Complex pathways and memory in compressed corrugated sheets. *Proc. Natl. Acad. Sci.* **118**, e2111436118 (2021).
10. MM Terzi, M Mungan, State transition graph of the preisach model and the role of return-point memory. *Phys. Rev. E* **102**, 012122 (2020).
11. M Mungan, MM Terzi, The structure of state transition graphs in systems with return point memory: I. general theory. *Annales Henri Poincaré* **20**, 2819–2872 (2019).
